# Cardiac and Gastric Interoceptive Awareness Have Distinct Neural Substrates

**DOI:** 10.1523/ENEURO.0157-22.2023

**Published:** 2023-01-27

**Authors:** Yusuke Haruki, Kenji Ogawa

**Affiliations:** Department of Psychology, Graduate School of Humanities and Human Sciences, Hokkaido University, Sapporo 060-0810, Japan

**Keywords:** interoceptive awareness, interoceptive attention, cardiac interoception, gastric interoception, fMRI, insula

## Abstract

Interoceptive awareness, an awareness of the internal body state, guides adaptive behavior by providing ongoing information on body signals, such as heart rate and energy status. However, it is still unclear how interoceptive awareness of different body organs are represented in the human brain. Hence, we directly compared the neural activations accompanying attention to cardiac (related to heartbeat) and gastric (related to stomach) sensations, which generate cardiac and gastric interoceptive awareness, in the same population (healthy humans, *N* = 31). Participants were asked to direct their attention toward heart and stomach sensations and become aware of them in a magnetic resonance imaging (MRI) scanner. The results indicated that the neural activations underlying gastric attention encompassed larger brain regions, including the occipitotemporal visual cortices, bilateral primary motor cortices, primary somatosensory cortex, left orbitofrontal cortex, and hippocampal regions. Cardiac attention, however, selectively activated the right anterior insula extending to the frontal operculum compared with gastric attention. Moreover, our detailed analyses focusing on the insula, the most relevant region for interoceptive awareness, revealed that the left dorsal middle insula encoded cardiac and gastric attention via different activation patterns, but the posterior insula did not. Our results demonstrate that cardiac and gastric attention evoke different brain activation patterns; in particular, the selective activation may reflect differences in the functional roles of cardiac and gastric interoceptive awareness.

## Significance statement

Interoceptive awareness, senses that arise from within the body, play a critical role in adaptive behavior by providing ongoing information on bodily states, such as the heart rate and energy status. Although interoceptive awareness has various functions depending on its source, previous neuroimaging studies have extensively used cardiac awareness (related to the heartbeat). The present study showed that attention to cardiac and gastric (related to the stomach) sensations evoked distinct neural activation patterns by combining mass-univariate analysis with multivoxel pattern analysis (MVPA) using functional magnetic resonance imaging (fMRI), indicating that the brain encodes attention to and thus awareness of different bodily organs in different manner. Moreover, the selective brain activation may reflect differences in the functional roles of cardiac and gastric awareness.

## Introduction

Subjective experiences of internal bodily states are referred to as interoceptive awareness (Khalsa et al., 2018), and such senses indicate ongoing physiological changes and guide adaptive behavior (Paulus et al., 2019; Quigley et al., 2021). Although interoceptive awareness serves multiple functions, depending on its source, e.g., thirst, hunger, heartbeat perception, it remains unclear how subjective differences in interoceptive awareness are represented in the human brain (Azzalini et al., 2019). We used functional magnetic resonance imaging (fMRI) to focus on the brain activations accompanying attention to cardiac (related to the heartbeat) and gastric (related to the stomach) sensations that elicit interoceptive awareness of different body organs, which have been suggested to serve different functional roles.

Considering the types of situations where people become aware of their heartbeat, the heartbeat signals and cardiac interoceptive awareness inform changes in physiological arousal (Paulus et al., 2019). In experimental settings, accelerated false cardiac feedback, i.e., exaggerated cardiac interoceptive awareness, has been found to alter the emotional salience of neutral faces (Gray et al., 2007) and perceived physical effort (Iodice et al., 2019) by enhancing physiological arousal. Similarly, gastric interoceptive awareness, such as fullness or hunger, modulates foraging and feeding behavior. In fact, people with eating disorders show altered gastric function and interoceptive awareness (Walsh et al., 2003; van Dyck et al., 2021), which may cause their abnormal eating behavior. Despite considerable differences in their functional roles, extant research has failed to establish whether cardiac and gastric interoceptive awareness are differently represented in the brain.

The invasive procedures needed to manipulate bodily signals have limited detailed functional brain mapping of interoceptive awareness. The brain regions responding to changes in visceral signals and interoceptive awareness have been studied using physical stimulation of viscera (Lu et al., 2004; Jarrahi et al., 2015) or pharmacological disturbance (Hassanpour et al., 2016, 2018) during fMRI scanning. However, such paradigms are difficult to combine in a single study because of invasiveness and the requirement of specific instruments, e.g., a barostat or an intravesical infuser. In addition, these brain activations would reflect the participant’s discomfort and anxiety, induced by the abnormal sensations, and thus may not be appropriate for mapping interoceptive awareness of different organs. Another way to map interoceptive awareness is to use an interoceptive attention paradigm, asking participants to direct their attention toward sensations originating from the body. Importantly, although exteroceptive information, such as cutaneous sensations, may contribute to interoceptive awareness (Khalsa et al., 2009), interoceptive attention has been found to elicit interoceptive awareness and robust activations of brain regions, including the insula, middle cingulate cortex, and supplementary motor area (Caseras et al., 2013; Tan et al., 2018; Haruki and Ogawa, 2021). In particular, researchers have consistently associated activation of the right anterior insula with interoceptive attention (Zaki et al., 2012; Simmons et al., 2013) and objective accuracy of heartbeat perception (Critchley et al., 2004; Pollatos et al., 2007; Caseras et al., 2013). These results appear to support an influential neuroanatomical model of interoceptive awareness, where the posterior insula receives the initial cortical input of visceral signals while awareness of internal bodily states is represented in the right anterior insula (Craig, 2009, 2011; Evrard, 2019).

Previous studies using the interoceptive attention paradigm suffer from the limitation of having extensively used heartbeat attention, eliciting cardiac interoceptive awareness, over other bodily sensations. Even when attentional focus on gastric sensations was deployed, direct comparison within the modality of interoceptive attention has not been discussed (Simmons et al., 2013; Kerr et al., 2016; DeVille et al., 2020). Therefore, it is still unclear whether the neural encodings of interoceptive awareness of different bodily signals differ, limiting theoretical advances in this field. To address this issue, we directly compared the neural activations for cardiac and gastric interoceptive awareness using the interoceptive attention paradigm in a healthy population. We hypothesized that attention to cardiac and gastric sensations, and thus their interoceptive awareness, would elicit distinct neural activations, which would be modulated according to their functional roles. That is, cardiac attention might activate the regions underlying physiological arousal, while brain areas that modulate feeding and foraging behavior might show enhanced activation in gastric attention. Moreover, we considered that the insula, the most relevant region for interoceptive awareness (Craig, 2009; Critchley and Harrison, 2013), would show a subregion-specific representation of cardiac and gastric interoceptive awareness. To test this idea, we performed a region of interest (ROI) analysis, focusing on the insula, by combining multivoxel pattern analysis (MVPA) with a basic comparison of neural activation.

## Materials and Methods

### Participants

A total of 35 right-handed people participated in the experiment (15 women). One participant (a woman) requested an interruption of the experiment, yielding incomplete data. Of the remaining participants, the data of three (one man and two women) were excluded from the analysis because their maximum head movement was >3 mm during the experiment. Thus, the final analysis included 31 participants (12 women) who were 21.61 ± 2.45 years of age on an average (range: 20–31). Their handedness was assessed by a modified version of the Edinburgh Handedness Inventory for Japanese participants (Hatta and Nakatsuka, 1975). The sample size deemed sufficient for determining brain activation was based on previous studies that used a similar task for interoceptive attention (Wiebking et al., 2015; Haruki and Ogawa, 2021). Written informed consent was obtained from all participants. This study was conducted in accordance with the Declaration of Helsinki and all its amendments. The Ethics Committee of Hokkaido University approved the experimental protocol.

### Task procedures

**Figure 1.**
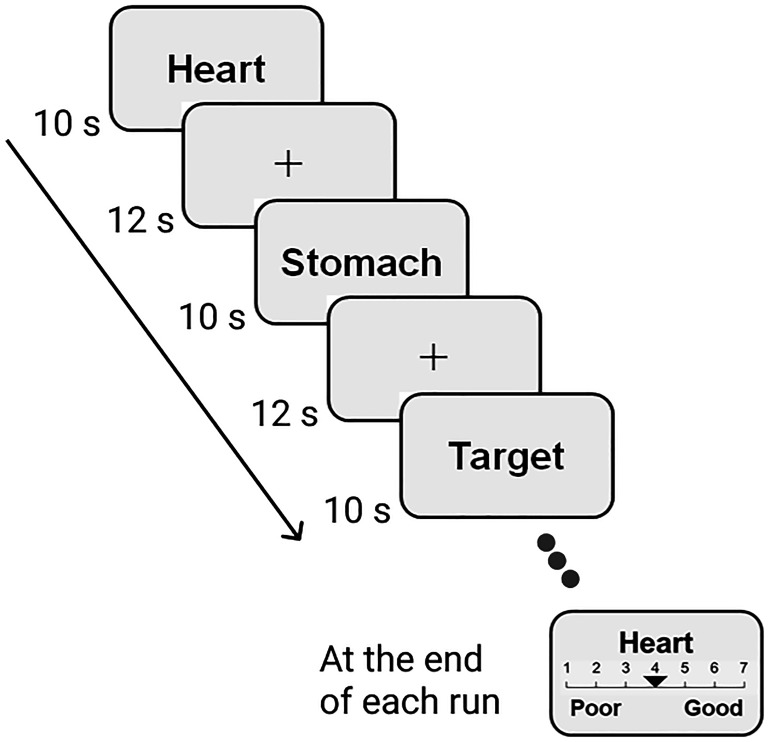
Schematic presentation of the task procedure. An example of the time course of the functional magnetic resonance imaging (fMRI) task is depicted. Our block design fMRI task included three types of task trials (heart attention, stomach attention, and control visual attention) that lasted 10 s each, followed by rest (12 s). Participants were asked to focus on sensations arising from the heart, stomach, and color changes in the word, according to the type of task. At the end of each run, participants rated the subjective intensity of the sensations for each condition using a Likert scale ranging from 1 (not intense at all) to 7 (extremely intense).

To quantify brain activation for cardiac and gastric interoceptive awareness noninvasively, we asked participants to perform an established interoceptive attention task in an MRI scanner. We used a task procedure that elicited robust brain activation for interoceptive awareness (Simmons et al., 2013; Kerr et al., 2016; DeVille et al., 2020), which was modified to suit our purpose, i.e., to perform MVPA, and to increase statistical power. The present task had a simple block design that had three conditions with resting periods: heart attention (for cardiac interoceptive awareness), stomach attention (for gastric interoceptive awareness), and visual attention (for control). Participants were asked to focus on sensations from their heartbeat or stomach, or visual stimuli, and to be aware of the slight sensations for each condition (Fig. 1). In the heart and stomach attention trials, the words “HEART” and “STOMACH” were presented for 10 s each on the screen to allow participants to realize which sensation they were focusing on. In the visual attention trial, the word “TARGET” was presented on the screen for 10 s, with the color of the word gradually and slightly fading from black to gray. The color changed every 1.5 s for a total of five times. Therefore, in each task trial, participants directed their attention to a particular, vague sensation without a salient stimulus. During the rest period, participants watched a fixation crossbar for 12 s with their eyes open. Preparing for the next task trial was prohibited.

Each condition of the task trial was presented five times per run: a single run included a total of 15 task trials and 15 rest periods. The order of the task trials was pseudo-randomized, but the same trial was not presented three times in succession. At the end of each run, participants rated the subjective intensity of the sensations (heart, stomach, and visual) throughout the run using a Likert scale ranging from 1 (not intense at all) to 7 (extremely intense). An ∼5.5-min run was repeated five times; thus, we obtained 25 data points (125 volumes) for each condition per participant. This procedure was designed to include a larger number of trials than those of previous studies (Kerr et al., 2016; DeVille et al., 2020) because we needed a sufficient number of task trials to perform MVPA. Before the first run began, all participants underwent a resting-state scan that lasted for 5 min, which was not analyzed in the present study. Stimuli were presented on a liquid crystal display and projected onto a custom-made viewing screen. Participants took a supine position in the scanner and viewed the screen via a mirror. Participants experienced a practice trial and filled its intensity report (for each condition), to learn how to perform the task and report the subjective intensity, before entering the MRI scanner. Through the practice trials, we verbally confirmed that the participants successfully directed their attention to each sensation depending on the condition.

### MRI acquisition

All scans were performed on a Siemens 3-Tesla Prisma scanner with a 64-channel head coil at Hokkaido University. T2*-weighted echoplanar imaging (EPI) was used to acquire a total of 168 scans per run, with a gradient echo EPI sequence. The first three scans within each session were discarded to allow for T1 equilibration. The scanning parameters used were as follows: repetition time (TR), 2000 ms; echo time (TE), 30 ms; flip angle (FA), 90°; field of view (FOV), 192 × 192 mm; matrix, 94 × 94; 32 axial slices; and slice thickness, 3.500 mm, with a 0.875-mm gap. Thus, the voxel size was 2.042 × 2.042 × 4.375 mm. T1-weighted anatomic imaging with an MP-RAGE sequence was performed using the following parameters: TR, 2300 ms; TE, 2.32 ms; FA, 8°; FOV, 256 × 256 mm; matrix, 256 × 256; 192 axial slices; and slice thickness, 1 mm without a gap.

### Preprocessing of fMRI data

All image preprocessing was performed using SPM12 software (Wellcome Department of Cognitive Neurology; http://www.fil.ion.ucl.ac.uk/spm). All the functional images were initially realigned to adjust for motion-related artifacts. Volume-based realignment was performed by co-registering images using rigid-body transformation to minimize the squared differences between volumes. The realigned images were then spatially normalized with the Montreal Neurologic Institute template based on the affine and nonlinear registration of co-registered T1-weighted anatomic images. They were resampled into 3 mm-cube voxels with sinc interpolation. The images were spatially smoothed using a Gaussian kernel of 6 × 6×6 mm full width at half-maximum. The images used for MVPA were not smoothed to avoid blurring the information in the multivoxel activity pattern.

### Statistical analysis

#### Subjective intensity of the sensations

We used R (version 4.0.3) for all our statistical inferences, except with functional imaging data. First, we tested whether the subjective intensity of the sensations differed between the modalities (cardiac, gastric, and visual). A linear mixed-effects (LME) model analysis, implemented in the lme4 package (Bates et al., 2015), was performed. With all the data obtained (*N* = 465, 3 trial types for 5 runs for 31 participants) as the dependent variable, we modeled the type of sensation as the fixed effect. Random intercepts and slopes for the effects of participants, and random intercepts for the runs, were modeled as random effects, ensuring the maximal random structure for our models (Barr et al., 2013). The LME allowed us to avoid averaging the values across five runs compared with the traditional ANOVA (Baayen et al., 2008). The parameters were estimated using the restricted maximum likelihood method, with the degrees of freedom estimated using the Satterthwaite method.

#### Whole-brain activation

We first evaluated the brain regions activated under each condition (heart attention, stomach attention, and visual attention) using a generalized linear model (GLM). An individual-level GLM included three regressors of interest for each condition as a separate box-car function that was convolved with the canonical hemodynamic response function. The rest period was used as a baseline. To reduce motion-related artifacts, six motion parameters were included as nuisance regressors. By combining the three conditions, we obtained six contrast images (heart attention compared with stomach attention, heart compared with visual, stomach compared with visual, and their opposite contrasts) for each participant. We then performed a group-level random effects analysis for these images using a one-sample *t* test. Through these statistical inferences, we directly compared the activation for each condition across the whole brain.

Moreover, we performed a group-level analysis of covariance (ANCOVA) to exclude the effect of the subjective ratings of stimulus intensity on brain activation. This was because we considered that the differences in subjective intensity could affect the brain activation pattern, independently of the object of attentional focus. Using the image of interoceptive attention, contrasted to the visual control, we modeled individual subjective ratings for each condition, averaged for five runs, as covariates. Then ANCOVA, excluding the effects of subjective rating as nuisance, was performed. By doing so, we assessed the brain activations of cardiac attention, contrasted with visual attention, without the effect of individual differences in subjective heartbeat intensity; and gastric attention, contrasted with visual, without the subjective intensity of stomach sensation. Furthermore, we explored variations in brain activation as a function of subjective intensity; a regression analysis with subjective intensity as a covariate of interest was performed using the same model used in the ANCOVA. For all the analyses, the voxel-level threshold was set to *p *<* *0.001 (uncorrected), and the cluster-level threshold was set to *p *<* *0.05, corrected for family-wise error (FWE).

#### ROI analysis of the subregions of the insula

We tested whether cardiac and gastric interoceptive awareness at the voxel level were differently represented in the subdivisions of the insula. In particular, we first performed MVPA, which allowed us to investigate more sophisticated neural representation than did conventional analysis (Norman et al., 2006), suspecting that conventional analysis might fail to detect activation differences in the insula. The ROIs were defined as the anatomic subdivisions of the insula in the Hammersmith brain atlases (Brain Development; www.brain-development.org). These images were constructed as a 3D probabilistic atlas using in vivo T1 MR images, including anatomic structures commonly seen in the human insula: the anterior short gyrus (ASG; the most dorsal anterior portion of the insula), middle short gyrus (MSG; the dorsal mid-anterior), posterior short gyrus (PSG; the dorsal mid-posterior), anterior inferior cortex (AIC; the ventral anterior), anterior long gyrus (ALG; the dorsal posterior), and posterior long gyrus (PLG; the ventral posterior; Faillenot et al., 2017). Importantly, these ROIs have been confirmed to show a subregion-specific activation pattern for heart attention (and, therefore, cardiac interoceptive awareness; Haruki and Ogawa, 2021).

The MVPA for heart and stomach interoceptive attention was performed with a two-class classifier based on a linear support vector machine (SVM) implemented in LIBSVM (http://www.csie.ntu.edu.tw/∼cjlin/libsvm/). We first created another individual-level GLM, apart from the whole-brain analyses that included 15 task trials as independent regressors per run with six motion parameters as nuisance regressors. By doing so, we obtained parameter estimates of all the voxels in each ROI, for a total of 75 trials per participant, labeling them as heart, stomach, or visual, depending on the condition of each trial. We then trained SVM to classify the identity of the brain activation pattern of heart and stomach interoceptive attention using these parameter estimates as inputs to the SVM. Individual-level classification accuracy was estimated with a 5-fold “leave-one-out” cross-validation to avoid overfitting. This procedure uses inputs in four runs as training data, and inputs in one remaining run as test data, which was repeated five times for all possible combinations. The averaged classification accuracy across five repetitions of the tests was computed for each ROI for each participant, independently. We used a default hyperparameter (a fixed regularization parameter C = 1). The parameter estimates of the trial were not used as inputs to the classifier. One-sample *t* tests were performed to test whether the activation patterns for heart and stomach interoceptive attention in each subregion were classifiable using SVM. That is, we tested whether the computed classification accuracy was higher than the chance level (50%) at the group level, separately for each ROI. Because we used 12 ROIs (six anatomic regions for both hemispheres), the calculated *p* values were corrected for Benjamini and Hochberg’s false discovery rate (FDR; Benjamini and Hochberg, 1995).

We also compared the differences in the averaged activations in the ROIs among each condition because we suspected that the classification accuracy calculated by MVPA could merely reflect the mean signal change in the ROIs. First, the mean signal changes in each ROI were extracted for each participant, separately for the heart and stomach attention conditions. Then, we performed a repeated-measures ANOVA on these values with the conditions (heart and stomach) and anatomic locations (the ASG, MSG, PSG, AIC, ALG, and PLG) as within-factors, independently for each hemisphere. Multiple comparison correction for *post hoc* analyses was performed using Shaffer’s modified sequentially rejective Bonferroni procedure implemented in R. The combined use of MVPA and direct comparisons of the averaged signal change allowed detailed investigation of how interoceptive awareness specific to the cardiac and gastric domains are represented in the human insula.

Furthermore, we tested whether the averaged activation for interoceptive and exteroceptive attention differed in the insula, using a similar procedure for cardiac and gastric interoceptive attention. We extracted mean signal changes for interoceptive attention, the averaged activation across heart and stomach attention, and exteroceptive attention (visual attention) in each ROI. Then repeated-measures ANOVA, with the condition (interoceptive and exteroceptive) and the anatomic location as within-factors, was performed separately for each hemisphere.

### Data availability

The .nii format figures of brain activation that support our findings are available at https://neurovault.org/collections/13262/. The .mat files containing the group-level brain activation, the results of our ROI analyses, and the behavioral data are also available at https://osf.io/8pg37/. The computer codes are available from the corresponding author upon reasonable request.

## Results

### Subjective intensity of the sensations

We performed an LME model analysis for the subjective ratings of the intensity of each sensation. The LME included the type of stimuli (heart, stomach, and visual) as the main factors; random intercept and slope for the effects on the participants and random slope for the sequence of runs, were modeled as random effects. The subjective intensity of visual stimuli was rated the highest (marginal mean contrasted to heart = 0.90, *t*_(30.00)_ = 4.51, *p *<* *0.001) and that of the stomach the lowest (marginal mean = −0.41, *t*_(30.00)_ = −2.22, *p *=* *0.03) among the three stimuli (Table 1).

**Table 1:** Subjective intensity of sensations in each condition

			95% CI for marginal means				
Predictor	Marginal mean	SE	Lower	Upper	e.d.f.	*t*	*P*
(Intercept)	4.71	0.16	4.33	5.09	31.00	24.69	<0.001
Stomach compared with heart	−0.41	0.18	−0.77	−0.04	30.00	−2.22	0.034
Visual compared with heart	0.90	0.20	0.50	1.29	30.00	4.51	<0.001

The linear mixed-effects model included the condition as the fixed effect. The random structure was maximal for appropriate inference: random intercept and slope for the effect of the participants, and random intercept for the effect of the run sequence, were modeled as random effects. SE, standard error for marginal means; CI, confidence interval; e.d.f., estimated degrees of freedom.

### Whole-brain activation for cardiac and gastric attention

**Figure 2.**
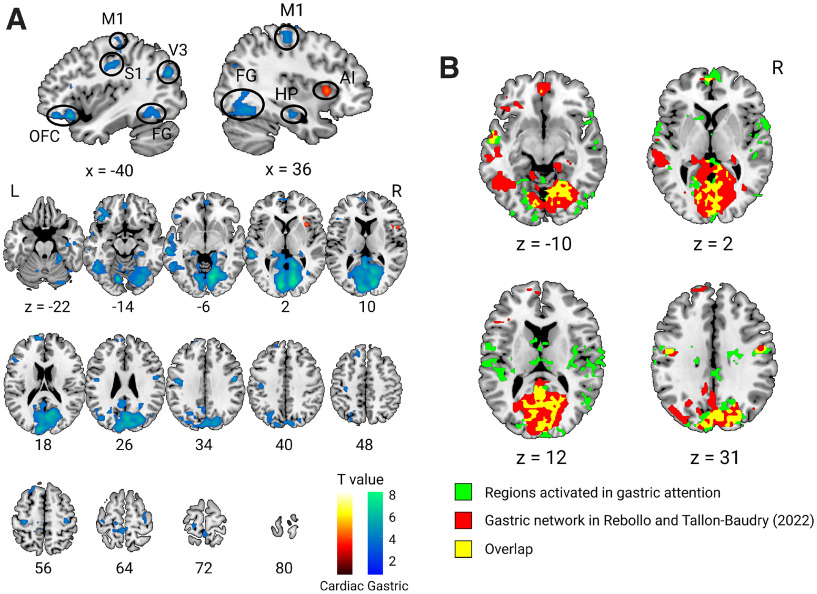
Whole-brain activation for cardiac and gastric attention. ***A***, Significant activation in cardiac attention contrasted with gastric attention (warm color) and vice versa (cold color) are depicted with a height threshold of *p *< 0.001 (uncorrected) and an extent threshold of *p* < 0.05 (corrected for family-wise error, corresponding to >42 voxels). Sagittal views show that attention to cardiac and gastric sensations activated the brain region relevant to each interoceptive awareness. The sensorimotor regions (S1, primary sensory; M1, primary motor; V3, visual area 3; FG, fusiform gyrus), orbitofrontal cortex (OFC), and hippocampus (HP) showed enhanced activation in gastric attention, while the right anterior insula (AI) was activated in cardiac attention. ***B***, The internally extended visual area that was activated in gastric attention largely overlapped with the brain regions that have been shown to couple with the gastric basal rhythm (Rebollo and Tallon-Baudry, 2022; https://neurovault.org/collections/9985/). L, left hemisphere; R, right hemisphere. All figures are shown in axial slices with z and sagittal slices with x denoting locations in the Montreal Neurologic Institute coordinates.

**Figure 3.**
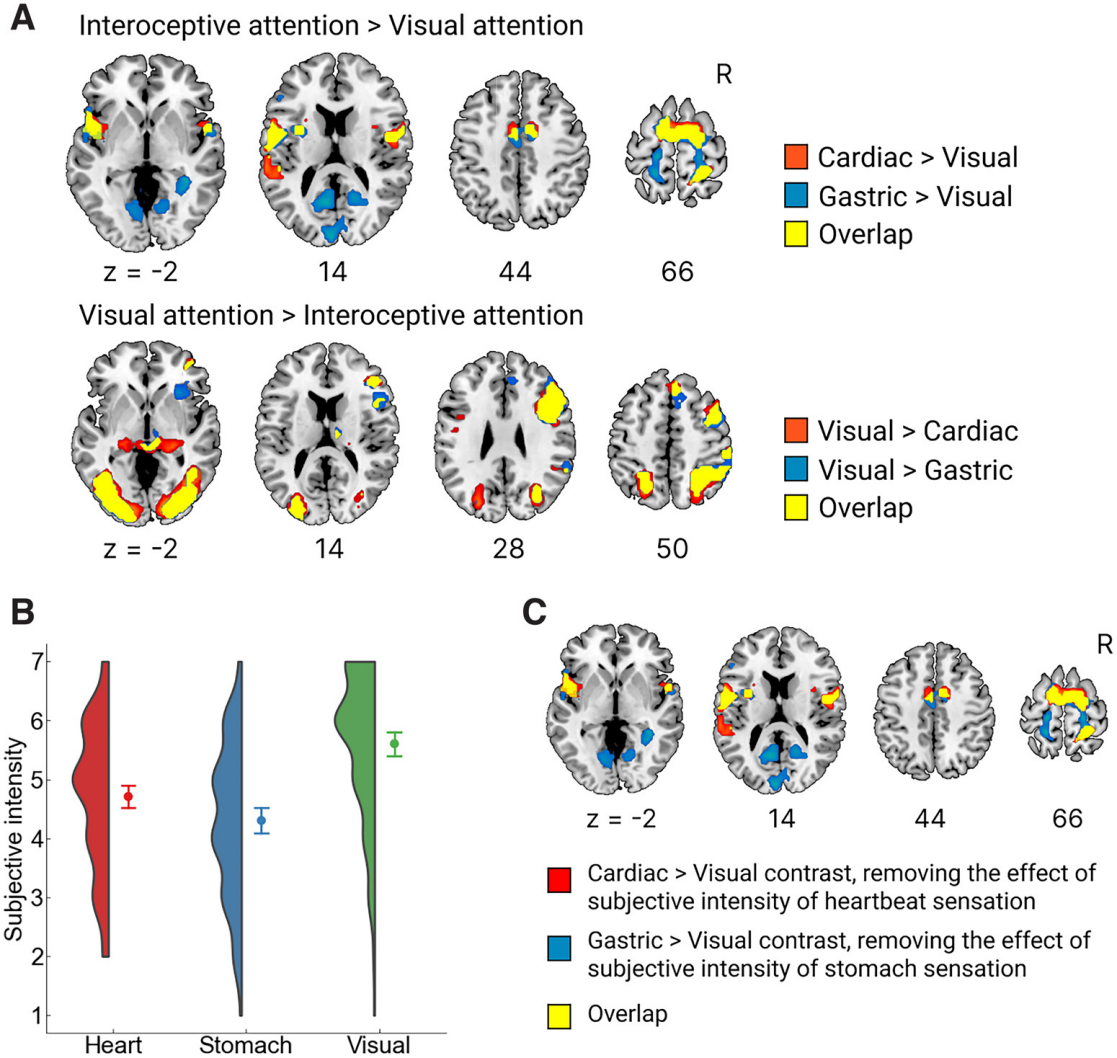
Brain activation for cardiac and gastric attention compared with the control. ***A***, When compared with the visual attention condition, cardiac and gastric attention showed similar activation patterns, such as activations in the insula and supplementary motor areas and deactivations in the right middle frontal, superior parietal, posterior thalamus, and lateral visual association areas. However, activations in the medial visual cortex and hippocampus were found only in gastric attention, compared with visual attention. The height threshold was set to *p* < 0.001 (uncorrected) with an extent threshold of *p* < 0.05 (corrected for family-wise error), corresponding to >52 voxels. ***B***, The distribution and mean scores of the subjective reports of stimulus intensity are plotted separately for each condition. Participants reported the intensity at the end of the scanning runs for each condition, yielding 155 datasets (31 participants for 5 runs) for each condition. Our maximal linear mixed-effects model analysis revealed that the visual stimulus (slight change in word color) was rated the most intense (regression coefficient compared with heart = 0.90, *t*_(30.00)_ = 4.51, *p *< 0.001) and the stomach sensation was rated the least intense (regression coefficient = −0.41, *t*_(30.00)_ = −2.22, *p *= 0.034). Error bars show the 95% confidence intervals, while the half-violin plot represents the kernel density estimation. ***C***, The brain activation underlying cardiac and gastric interoceptive awareness did not vary as a function of the subjective report of the stimulus intensity. We performed the analysis of covariance that excluded the effect of the subjective reports from brain activation, revealing almost the same activation patterns compared with the activations reported in ***A***. The height threshold was set to *p* < 0.001 (uncorrected) with an extent threshold of *p *< 0.05 (corrected for family-wise error), corresponding to >52 voxels. R, right hemisphere. All figures are shown in axial slices with z denoting locations in the Montreal Neurologic Institute coordinates.

We directly compared whole-brain activation across the three conditions (heart attention, stomach attention, and visual attention as the control). We found that stomach attention that elicited gastric interoceptive awareness activated larger brain areas, including the occipitotemporal visual cortices, bilateral primary motor, primary somatosensory, left orbitofrontal, and bilateral posterior hippocampus. In contrast, the right dorsal anterior insula extending to the frontal operculum only showed higher activation in heart attention, i.e., selectively for cardiac interoceptive awareness (Fig. 2*A*; Table 2). In particular, the medial visual area that was enhanced during gastric interoceptive attention largely overlapped with the “gastric network,” that showed a temporal coupling with the gastric basal rhythm (Fig. 2*B*; Rebollo and Tallon-Baudry, 2022). Moreover, by comparing the heart and stomach conditions to the visual control, we found that cardiac and gastric interoceptive attention activated similar brain regions, including the insula, frontal operculum, parietal operculum, middle cingulate, and supplementary motor area; the results were highly comparable to previous studies (Caseras et al., 2013; Tan et al., 2018; Haruki and Ogawa, 2021). A notable exception was that the hippocampus and medial visual areas were activated only in stomach attention (Fig. 3*A*). The visual control condition, contrasted with the heart and stomach conditions, activated brain regions critical for visual attention, such as the middle frontal gyrus, superior parietal lobule, posterior thalamus (geniculate nucleus), and lateral visual association area (Dosenbach et al., 2007; Mayer et al., 2007), supporting the validity of our experimental design (Fig. 3*A*). Furthermore, we confirmed that the brain activation elicited by cardiac and gastric interoceptive attention was almost unaffected by differences in subjective stimulus intensity by performing an ANCOVA excluding the effect of the subjective ratings of stimulus intensity. The results of the ANCOVA were fairly comparable to the brain activation obtained with cardiac and gastric interoceptive attention contrasted with that in visual control (Fig. 3*B*, 3*C*). There was no brain activation covarying with the subjective intensity rating even at a more moderate threshold (cluster-level *p *<* *0.05, uncorrected). All results are reported with a voxel-level threshold of *p *<* *0.001 (uncorrected) with cluster size correction for *p* values < 0.05 (FWE).

**Table 2:** Anatomical regions, peak voxel coordinates, and *t* values of observed activations

Location	Voxels	MNI coordinates			*t* value
		*x*	*y*	*z*	
Contrast: cardiac attention > gastric attention					
R dorsal anterior insula	48	33	17	2	4.65
Frontal operculum		54	11	8	4.39
Dorsal anterior insula		30	26	2	3.72
Contrast: gastric attention> cardiac attention					
L secondary visual area	3793	−12	−73	−13	8.42
R primary visual area		9	−82	5	8.29
Secondary visual area		18	−79	5	8.06
L middle temporal gyrus	211	−60	−31	−4	6.71
Superior temporal gyrus		−57	−7	−7	4.64
middle temporal gyrus		−54	−19	−7	4.29
L lateral orbitofrontal cortex	95	−42	26	−16	6.21
		−36	38	−16	5.29
		−51	26	−4	3.99
L primary motor cortex	67	−48	−7	29	5.90
R primary motor cortex	41	48	−7	35	5.88
L primary sensory area	116	−12	−25	59	5.50
		−3	−37	68	4.57
		−18	−37	68	4.26
L fusiform gyrus	181	−51	−58	−13	5.31
		−48	−46	−13	4.52
		−36	−61	−13	4.18
L primary motor cortex	47	−36	−25	53	5.18
		−39	−22	65	3.71
L dorsomedial prefrontal cortex	96	−12	62	17	5.14
		−18	59	29	5.00
		−9	59	5	4.93
L medial orbitofrontal cortex	52	−3	47	−13	5.11
		−3	56	−10	4.45
L frontal eye field	50	−21	32	53	5.05
		−12	35	44	4.72
R premotor area	50	33	−22	53	4.88
		33	−10	68	4.80
		33	−25	62	4.46
L dorsolateral prefrontal cortex	42	−54	26	23	4.47
		−39	26	14	3.98
		−48	29	5	3.72
Contrast: cardiac attention> visual attention					
L frontal operculum	578	−51	5	5	8.98
Dorsal anterior insula		−33	5	8	6.56
Supramarginal gyrus		−57	−31	26	6.54
R supplementary motor area	652	9	−1	68	8.90
L supplementary motor area		−9	−7	68	8.69
		18	−13	65	5.67
R frontal operculum	199	48	−1	8	8.81
		60	2	11	5.83
Dorsal anterior insula		36	8	11	5.09
R primary somatosensory area	112	24	−40	68	6.33
Contrast: gastric attention> visual attention					
R supplementary motor area	950	9	−7	59	8.45
L supplementary motor area		−9	−7	71	8.13
R primary somatosensory area		24	−40	68	7.41
L frontal operculum	490	−51	2	8	7.07
		−45	−4	2	6.51
Dorsal anterior insula		−33	2	11	6.06
R frontal operculum	151	48	−1	8	7.02
		60	2	11	5.28
		60	5	20	5.21
R posterior hippocampus	94	33	−40	−4	6.55
		33	−49	2	5.09
		36	−31	−10	4.70
R secondary visual area	1011	12	−88	23	6.48
L secondary visual area		−6	−94	20	5.97
		0	−85	23	5.83
L angular gyrus	161	−48	−73	29	5.87
		−36	−61	23	5.20
		−36	−82	35	4.66
R visual association area	63	18	−61	−4	4.34
		21	−64	−16	3.58
Contrast: visual attention > cardiac attention					
R visual association area	4302	33	−82	−7	10.86
Secondary visual area		27	−91	2	10.27
L secondary visual area		−33	−91	2	10.04
R frontal operculum	849	45	11	29	7.73
Premotor area		45	5	53	6.79
Dorsolateral prefrontal cortex		45	35	20	5.81
R dorsal posterior thalamus	309	18	−31	2	6.72
		−24	−28	−1	5.52
		9	−28	−1	5.52
R medial orbitofrontal cortex	132	21	32	−13	6.58
Frontal pole		36	50	−10	5.36
Lateral orbitofrontal cortex		45	41	−13	4.37
L primary motor area	52	−45	−10	35	5.13
		−45	5	32	4.70
R frontal eye field	116	3	35	47	5.00
		6	26	56	4.88
Contrast: visual attention > gastric attention					
R fusiform gyrus	1495	27	−79	−1	10.36
Visuomotor area		27	−49	44	9.02
Secondary visual area		24	−88	−4	8.66
L secondary visual area	812	−33	−91	2	9.88
		−27	−91	−4	9.43
		−27	−97	8	7.86
R premotor area	996	45	2	53	7.99
Frontal operculum		45	11	29	7.83
Dorsolateral prefrontal cortex		45	35	20	5.96
L cerebellum	204	−9	−76	−40	6.94
		−6	−76	−31	5.90
		−6	−73	−19	5.38
R dorsal thalamus	124	6	−13	5	6.80
		6	−28	−1	5.53
L superior parietal cortex	149	−24	−61	56	6.56
		−24	−61	47	5.95
R frontal eye field	173	12	35	38	5.61
		9	23	56	5.58
		6	29	44	4.89
L cerebellum	65	−15	−40	−43	5.28
		3	−31	−31	4.77
		−9	−37	−37	4.43
R frontal pole	52	39	44	−4	4.42
		36	53	−7	4.41

The peak-level threshold was set to *p *<* *0.001, and the cluster size was also corrected for an extent threshold of *p *<* *0.05 (corrected for family-wise error). L, left hemisphere; MNI, Montreal Neurologic Institute; R, right hemisphere.

### Multivoxel pattern classification for cardiac and gastric attention in the insula

**Figure 4.**
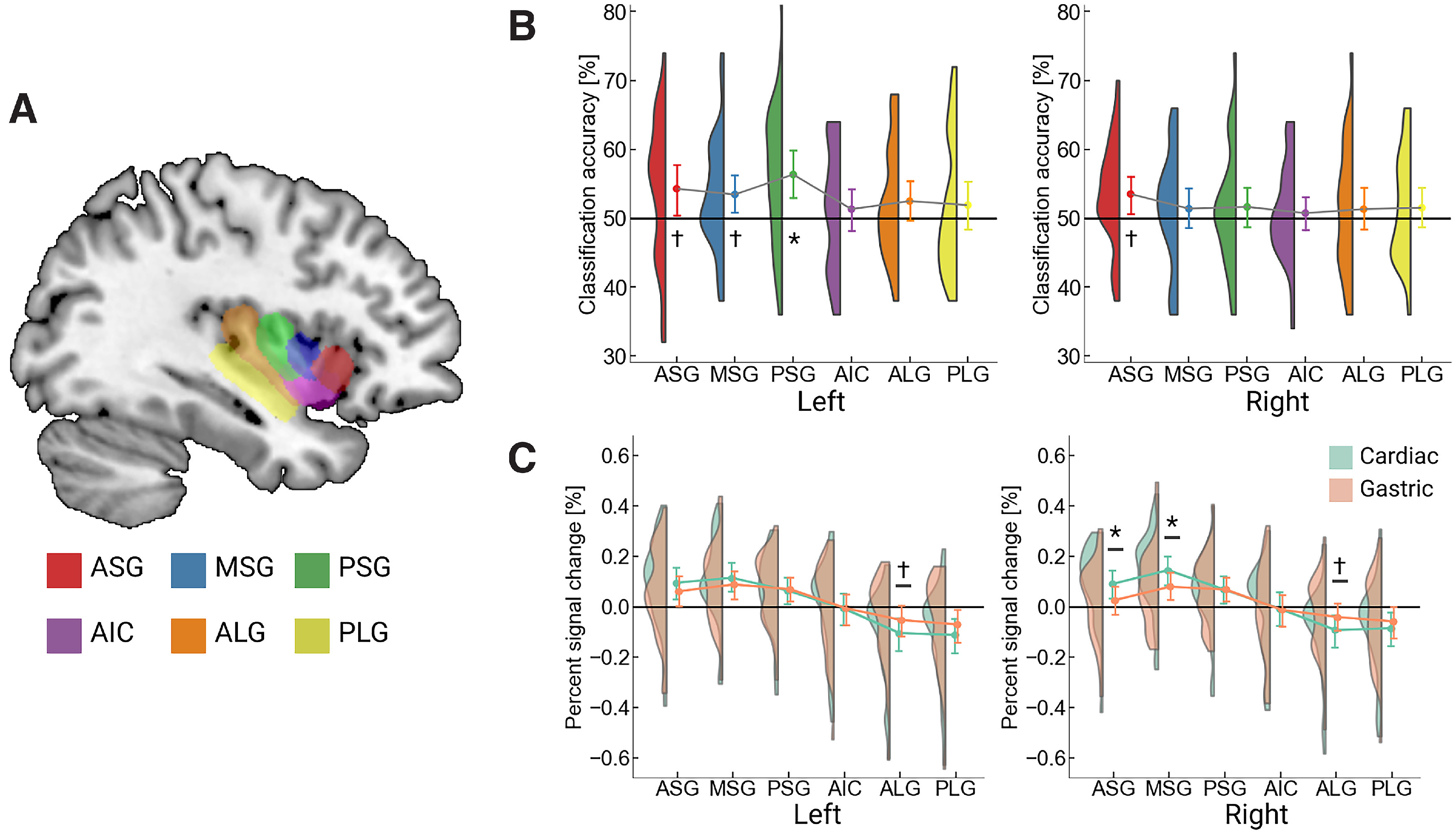
Comparison of activations for cardiac and gastric attention in the subdivisions of insula. ***A***, The anatomic subdivisions of the insula (Faillenot et al., 2017) are presented. We adopted six regions (ASG, anterior short gyrus; MSG, middle short gyrus; PSG, posterior short gyrus; AIC, anterior insular cortex; ALG, anterior long gyrus; PLG, posterior long gyrus) for both hemispheres. ***B***, The results of multivoxel pattern analysis are depicted, with the left panel showing the results of the left insula subdivisions and the right panel showing the right insula. The left MSG exhibited significantly higher classification accuracy above the 50% chance level. A marginally significant classification accuracy, i.e., *p *< 0.1, was observed in the right ASG, left ASG, and left MSG. The point plot represents the mean classification accuracy with a 95% confidence interval, while the half-violin plot represents the kernel density estimation. ***C***, The results of direct comparison of the signal strength between cardiac and gastric attention are plotted. *Post hoc* analysis of repeated-measures ANOVA revealed that the right ASG and MSG showed significantly higher activation in cardiac than gastric attention. There was a marginally significant effect that implies stronger activation in gastric than cardiac attention in the left ALG and right ALG. The point plots represent the mean signal change, with 95% confidence interval, while the half-violin plot represents the kernel density estimation (green for cardiac, orange for gastric attention). *p < 0.05; †*p* < 0.10 (corrected for false discovery rate). As Extended Data, we performed a similar analysis focusing on the subdivisions of the insula for averaged brain activation in interoceptive and exteroceptive attention (Extended Data Fig. 4-1).

We performed MVPA using an SVM classifier that distinguished the activation patterns in the insula in heart and stomach conditions. Activation patterns in the anatomic subregions of the insula (Faillenot et al., 2017; Fig. 4*A*) were used as inputs to the SVM. We found that classification accuracy in the left PSG, which corresponds to the dorsal middle insula, was significantly higher than the chance level (50%; mean classification accuracy = 56.32, *t*_(30)_ = 3.39, *d *=* *0.61, corrected *p*-value for FDR = 0.024), suggesting that the region had distinct representation for cardiac and gastric interoceptive awareness. The right ASG (mean = 53.48, *t*_(30)_ = 2.56, *d *=* *0.46, corrected *p *=* *0.089), left ASG (mean = 54.26, *t*_(30)_ = 2.28, *d *=* *0.41, corrected *p *=* *0.089), and left MSG (mean = 53.42, *t*_(30)_ = 2.34, *d *=* *0.42, corrected *p *=* *0.089) showed a marginally significant classification accuracy above the chance level; all corresponded to the dorsal mid-anterior insula. The other subdivisions of the insula, such as the posterior or ventral subdivisions, showed no significant classification accuracy above the chance level (corrected *p*s* *>* *0.27, *d*s* *<* *0.30; Fig. 4*B*).

10.1523/ENEURO.0157-22.2023.f4-1 Extended Data Figure 4-1 Comparison of activations for interoceptive and exteroceptive attention in the subdivisions of the insula. The results of direct comparison of the signal strength between interoceptive (averaged for heartbeat attention and stomach) and exteroceptive (visual) attention are plotted. *Post hoc* analysis of repeated-measures AMOVA revealed that the right ASG showed significantly higher activation in exteroceptive than interoceptive attention, while the left MSG and PSG were activated more in interoceptive attention. The point plots represent the mean signal change, with 95% confidence intervals, while the half-violin plots represent the kernel density estimation (green for interoceptive attention, orange for exteroceptive attention). Download Figure 4-1, TIF file.

### Comparison of the signal change for cardiac and gastric attention in the insula

We directly compared the signal change in the insular subregions for cardiac and gastric interoceptive attention. Averaged parameter estimates were extracted from the insular ROIs independently for heart and stomach conditions, i.e., 12 values for each condition. We then conducted a repeated-measures ANOVA, with the anatomic location and condition as the within-factors, independently for each hemisphere. The results indicated that, in both hemispheres, the main effects of anatomic location and the interaction between location and condition were significant (left, *F*_(5,150)_ = 39.18, η^2^*_p_* = .57, η^2^*_p_* = 0.65, *F*_(5,150)_ = 8.87, η^2^*_p_* = 0.22; right, *F*_(5,150)_ = 39.18, *F*_(5,150)_ = 15.42, η^2^*_p_* = 0.34; all *p*s* *<* *0.001), but the main effect of the condition was not (left, *F*_(1,30)_ = 0.07, η^2^*_p_* = 0.00; right, *F*_(1,30)_ = 0.15, η^2^*_p_* = 0.01; *p *>* *0.70; Fig. 4*C*), replicating the results of a previous study (Haruki and Ogawa, 2021). *Post hoc* analysis of the left ROIs revealed that the ALG (corresponding to the dorsal posterior insula) showed a marginally significant activation that was higher for gastric attention than for cardiac attention (*F*_(1,30)_ = 3.83, corrected *p *=* *0.059). *Post hoc* analysis of the right ROIs also revealed that the right ASG and MSG exhibited higher activation for cardiac attention than for gastric attention (*F*_(1,30)_ = 5.93, corrected *p *=* *0.021; *F*_(1,30)_ = 6.57, corrected *p *=* *0.016, respectively) while a marginally significant activation, higher for gastric attention, was found in the ALG (*F*_(1,30)_ = 4.13, corrected *p *=* *0.051).

Furthermore, we found differences in the averaged activation between interoceptive and exteroceptive attention; the ANOVA on activations in the left insula revealed significant effects of anatomic location (*F*_(5,150)_ = 52.71, *p *<* *0.001, η^2^*_p_* = 0.64), and the interaction between condition and location (*F*_(5,150)_ = 13.45, *p *<* *0.001, η^2^*_p_* = 0.31; Extended Data Fig. 4-1). *Post hoc* analysis showed that the left MSG and PSG were more activated by interoceptive attention (*F*_(1,30)_ = 7.38, *p *=* *0.011; *F*_(1,30)_ = 18.70, *p *<* *0.001, respectively). The ANOVA on the right insula activation revealed significant effects from anatomic location (*F*_(5,150)_ = 41.02, *p *<* *0.001, η^2^*_p_* = 0.58), and interaction between condition and location (*F*_(5,150)_ = 12.23, *p *<* *0.001, η^2^*_p_* = 0.28), as well. We found that exteroceptive attention activated the right ASG more than interoceptive attention did, by *post hoc* analysis (*F*_(1,30)_ = 10.28, *p *=* *0.003).

## Discussion

Our results empirically showed that cardiac and gastric interoceptive awareness had similar but distinct neural substrates. Direct comparisons of brain activations revealed that interoceptive attention to heart and stomach sensations activated similar brain regions, including the insula, frontal operculum, parietal operculum, middle cingulate, and supplementary motor area, in contrast to the control visual attention. However, compared with gastric attention, cardiac attention activated the right anterior insula extending to the frontal operculum more; gastric attention enhanced activations in the occipitotemporal visual cortices, bilateral primary motor, primary somatosensory, left orbitofrontal, and bilateral posterior hippocampus. Moreover, our MVPA-based ROI analyses revealed that the left middle-anterior insula had distinct neural representations for cardiac and gastric interoceptive attention.

Previously, heartbeat perception has been studied as a representative of interoceptive awareness in general, suggesting the right anterior insula as the most relevant region for the generation of subjective experiences of internal bodily states (Craig, 2009; Critchley and Garfinkel, 2017). We, however, found that cardiac attention activated the right anterior insula more than gastric attention did, suggesting that the right anterior insula may predominantly code cardiac interoceptive awareness, rather than interoceptive awareness arising from other sources, e.g., gastric. In line with this idea, the most consistent activation in the right insula has been found by a meta-analysis focusing on cardiac interoceptive attention (Schulz, 2016). Furthermore, previous studies have found a robust correlation between individual accuracy of heartbeat perception and heightened activation of the right anterior insula (Critchley et al., 2004; Pollatos et al., 2007; Caseras et al., 2013; Haruki and Ogawa, 2021), but this was not the case for awareness of breathing (Wang et al., 2019) or skin conductance (Baltazar et al., 2021). The activation dominance of the right anterior insula may be explained by the functional roles of cardiac interoceptive awareness: people can notice increased arousal with heartbeat perception (Paulus et al., 2019). The right insula appears essential to perceive arousal as resection of the region causally diminishes physiological and emotional arousal (Terasawa et al., 2021; Holtmann et al., 2022). Moreover, presenting accelerated cardiac feedback increases perceived physiological arousal (Story and Craske, 2008) and, more importantly, activates the right anterior insula (Gray et al., 2007; Kleint et al., 2015). These findings, in conjunction with the present results, imply that cardiac interoceptive awareness closely involves physiological arousal and thus activates the right anterior insula by merely focusing on cardiac sensation. To sum up, the relationship between the right anterior insula and interoceptive awareness appears stronger in cardiac interoceptive awareness than in other modalities; such dominance of the cardiac domain may reflect the functional role of cardiac interoceptive awareness, which informs physiological arousal.

We also found that the brain regions underlying gastric interoceptive awareness encompassed the visual cortex. The involvement of the visual cortices in attention to stomach sensations appears puzzling, but converging evidence suggests a strong connection between the visual cortex and stomach function. Recently, Rebollo and colleagues found temporally delayed connectivity between brain activity and the intrinsic electrical rhythm generated by the stomach, which included the occipitotemporal visual cortices in addition to the somatosensory and motor areas (Rebollo et al., 2018; Rebollo and Tallon-Baudry, 2022). Crucially, the visual areas activated in the current experiment largely overlapped with the clusters included in the gastric network. Other past research showed that electrical or vibration stimuli on the stomach evoked neural activation in the occipital area in rats (Cao et al., 2019), cats (Pigarev et al., 2013), and even humans (Mayeli et al., 2021). Considering all these reports, it can be suggested that gastric functions are strongly tied to the visual cortex; here, we demonstrated that interoceptive attention to the stomach, which elicits gastric interoceptive awareness, activates the visual cortex even without direct stimulation of the stomach.

The reason for the involvement of the visual area may be that gastric signaling, and its subjective awareness, are closely related to foraging and feeding behavior that requires the integration of visuospatial information with energy status and motor coordination (Kanoski and Grill, 2017; Rebollo et al., 2021). For example, hungry people would change their sensory sampling and behavior in the external world to maximize the chance of food consumption. The current results support this idea; we observed activation related to gastric attention in the left orbitofrontal cortex, bilateral hippocampus, primary motor cortex, and the visual areas, which are all associated with food intake. For example, the orbitofrontal cortex has been suggested to modulate eating behavior by encoding the nutritional and reward values for food or food cues (Seabrook and Borgland, 2020). A meta-analysis of fMRI studies, including viewing food pictures compared with nonfood pictures, indicated that the left orbitofrontal cortex was most consistently activated for visual food stimuli (van der Laan et al., 2011). Furthermore, the role of the hippocampus in controlling food intake and regulating energy status has received considerable attention in the past few years (Suarez et al., 2019; Quigley et al., 2021); recent evidence indicates that the human hippocampus encodes ongoing nutritional states in response to food cues (Jones et al., 2021). Based on these findings, we consider that neural responses to gastric interoceptive awareness could be encoded in relation to food intake, which was activated by focusing on stomach sensations without any stimulation or food-related cues in the present study.

The present ROI analyses revealed that cardiac and gastric interoceptive attention had distinct representations in the insula. Only the right ASG and MSG, corresponding to the dorsal anterior insula, showed higher activation in cardiac than gastric attention; in the right mid-posterior and left insula, there was no activation difference. Nevertheless, we identified the left PSG, corresponding to the dorsal middle insula, in different activation patterns for cardiac and gastric attention. These results elaborate on how interoceptive awareness is represented in the human insula; in particular, we suggest, for the first time, that the left middle insula codes viscera-specific interoceptive awareness. Interestingly, the posterior insula did not show separable activation for cardiac and gastric attention, nor higher activation than baseline. Researchers have considered the posterior insula as the “primary” interoceptive cortex as it receives an initial cortical input of visceral signals (Craig, 2002; Evrard, 2019). The current inactivity of the posterior insula may support its role in encoding ongoing changes in bodily signals (Craig et al., 2000; Meier et al., 2018), rather than a subjective awareness of bodily states. This is because, in the interoceptive attention task, participants must have interoceptive awareness without homeostatic perturbation. Together, our ROI analyses would support the gradual processing of bodily signals in the insula along the posterior-anterior axis (Craig, 2009): the posterior codes physical changes in the signal while interoceptive awareness would be represented in the middle-anterior insula.

Unfortunately, we did not record any physiological data during the fMRI scanning, potentially limiting the current study. Simultaneous recording of electrocardiograms (ECGs) and electrogastrograms would address the interaction between brain activation and physiological changes evoked by interoceptive attention. However, we believe the lack of physiological recording does not challenge the validity of the present study because past research indicated that cardiac parameters (heart rate and ECG amplitude) did not differ by directing attention between interoceptive and exteroceptive signals (Petzschner et al., 2019). On a related note, previous studies suggested that external processes, such as cutaneous sensations, could affect interoceptive awareness (particularly in the cardiac domain; Khalsa et al., 2009). Although we asked participants to focus on their internal sensations, we could not completely rule out the possibility that participants felt the heartbeat sensation from their skins. However, in the first place, interoceptive awareness may not arise solely from a single visceral sensation, such as when heartbeat perception is felt with the contraction of blood vessels and beating of the heart or hunger is mediated by both the physical contents of the stomach and a chemical signal. If so, researchers may benefit from isolating the neural substrates of cardiac and gastric interoceptive awareness localized in the present study from visceral topography to reveal the process of (visceral) multisensory integration. Another possible limitation is that the difference in task difficulty in each condition could affect the results. Although we tried to make the difficulty comparable for each condition, the task condition had a significant effect on the subjective intensity of stimulus; the subjective intensity of the visual stimulus was rated the highest while that of the stomach sensation was the lowest. We therefore performed another analysis that excluded the effect of the subjective rating of stimulus intensity, finding that brain activation was almost unaffected by individual differences in the subjective ratings. The results support our original idea that the present brain activation was elicited by the subjects (the heartbeat and stomach sensations) of attentional focus not by the difference in task difficulty. Recording a trial-by-trial fluctuation of the subjective intensity of stimuli would strengthen the present findings that the subjective differences in interoceptive awareness are encoded differently in the brain.

All the participants of this study were healthy adults aged between 20 and 31 whose native language was Japanese. However, we consider the present results to be replicable in other countries in healthy young adults because the brain activations induced by cardiac attention were comparable to that of previous studies conducted in Europe and the US (Caseras et al., 2013; DeVille et al., 2020). The stimulus used in the present experiment was a single word in English; therefore, we expect the results to be generalizable to healthy young adults as long as they understand the meaning of the word. The participants performed the task while they were lying in a supine position in an MRI scanner. Thus, it could be argued that the differences in brain activation of cardiac and gastric attention may be specific to interoceptive awareness in a supine position. We have no reason to believe that the results depend on other characteristics of the participants, materials, or context.

## Synthesis

Reviewing Editor: Christophe Bernard, INSERM & Institut de Neurosciences des Systèmes Decisions are customarily a result of the Reviewing Editor and the peer reviewers coming together and discussing their recommendations until a consensus is reached. When revisions are invited, a fact-based synthesis statement explaining their decision and outlining what is needed to prepare a revision will be listed below. The following reviewer(s) agreed to reveal their identity: Ignacio Rebollo. Note: If this manuscript was transferred from JNeurosci and a decision was made to accept the manuscript without peer review, a brief statement to this effect will instead be what is listed below.

Dear authors,

Thanks for your work on the neural correlates of gastric and cardiac interoception. The study is timely and informative, yet there are some important issues to be addressed before we can recommend it for publication. These entail, overall, wording (accuracy), analyses (transparency and further analyses that account for behavioral variability), and further discussion. We detail our comments in the following:

1. The term interoception is used too loosely and is difficult to interpret. While the authors state in the introduction that they refer to ‘the awareness of signals that arise from within the body as interoception’, in the rest of the manuscript they use vague terms such as ‘cardiac and gastric interoception’, ‘different interoceptions’, etc. What type of interoception are the authors measuring with the task? How does the task relate to interoceptive attention, sensitivity, subjective awareness or signal intensity? Please refer to recent literature on cardiac interoception (e.g., Khalsa et al 2018) and, once the authors have settled on a specific interpretation of the term, they should use it consistently throughout the manuscript.

2. The authors asked participants to attend to sensations of their heart and stomach. While these sensations might originate within the body, the process of such information might reach participants’ sensorirum/awareness through exteroceptive modalities. For instance, heartbeats produce a pulsatile sensation that can be felt on the chest over the skin (a cutaneous stimulation). See for instance the studies reviewed here: https://www.sciencedirect.com/science/article/pii/S0010945222000685 (second paragraph in the introduction and those also reported to show small movements concomitant to heartbeats), see also: https://www.nature.com/articles/nn.2411. Hence, while changes inside the body are interoceptive in origin and principle, conscious awareness of these changes might entail exteroceptive processing. This idea should be more clearly stated in the manuscript.

3. Readers might appreciate seeing the difference in behavioral responses for cardiac, gastric and visual task in a figure. Are they statistically different? These results are not reported and are important to interpret brain data. Please provide descriptive and inferential statistics for behavioral data in full (see also point 8). Also, the cardiac signal is typically felt more strongly than gastric sensations during rest. Without an external manipulation of gastric and cardiac signals, the difference in result could reflect the difference in signal strength, attentional demand, fatigue and “on task” vs “off task”. Regarding the limitations of the task, they should be addressed more lengthly in the discussion. What are the task confounds, which other variables could be measuring?

4. There is no analysis comparing with the visual condition, why is this so? The visual condition is a valid control that does not seem to be used at all. At the very least, the authors should report results of the contrasts gastric > visual and cardiac > visual as well as their reverse for brain data and, as pointed out in the previous comment, report the full behavioral results. Furthermore, in MVPA analyses the authors could identify insula regions associated with cardiac and gastric interoception over and above the visual control condition.

5. The MVPA analyses should be explained more clearly in both the results and methods sections, as at present it is unclear what the procedure and rationale of the analysis is. What conditions, exactly, were compared in each step, how, and why?

6. Direct heart rate or gastric cycle measurements would have greatly strengthened the study, as it would have allowed to measure not only where subjects’ attention is directed, but also how this relates to objective changes in cardiac or gastric rhythms (e.g., whether higher intensity ratings correspond with accelerations in heart rate). Are these measurements available? If not, this should be discussed as a major limitation, and studies using objective measurements should be clearly distinguished from the present one in the introduction (see also point 1).

7. The authors did not analyze how changes in bold signal vary as a function the subjects’ reports of sensations intensity. We suggest the authors to run both within-subject (parametric modulation with trial-wise intensity ratings) and between-subject (regression with mean intensity reports) analyses, reporting the results separately for each condition, and comparing the conditions between them. These analyses will strongly improve the paper.

8. On a related note, the results of the linear mixed effects models should be reported in full, including degrees of freedom, regression coefficients, and p-values. Also, please ensure that the random effects structure is maximally specified: https://www.ncbi.nlm.nih.gov/pmc/articles/PMC3881361/ Furthermore, raincloud plots should be used to display intensity ratings per condition (as suggested in point 3).

9. The overlap with the gastric network is not actually quantified, please provide a standard metric in this regard. Also, the authors should considering using the overlays from the most recent version of the gastric network from Rebollo & Tallon-Baudry (2022) https://identifiers.org/neurovault.collection:9985

10. Cardiac interoception. The article could benefit from some quantitative comparison with other studies of cardiac interoception. For instance, how the heart cluster relates to neurosynth studies of the term interoceptive (https://neurosynth.org/analyses/terms/interoceptive/) or heart (https://neurosynth.org/analyses/terms/heart/). Moreover, the scope of the discussion on cardiac interception should be limited. This is mainly because the limitations of the task in measuring more specific components of interoception. In particular, the fifth paragraph of the discussion should be restructured. The messages are difficult to extract and is generally difficult to read.

11. From a subject perspective, the authors should state that when attending to either stimuli (cardiac, gastric, visual), it is not possible to fully ignore sensations of the body. For instance, I could be attending to my stomach but noticing my heart. Please comment on this. Did the authors correct/dissociate for such an issue?

12. The authors did not report whether the number of trials and participants was appropriate and based upon prior evidence. The use of power calculation and/or citing papers with similar paradigms and analyses (as examples/guide) can aid to better buttress the design.

13. The last line of the first paragraph of the discussion is unclear, please reformulate

14. If possible the authors should share the data and code in an open repository. Please add the statement in the article about data availability, and if it is not possible, it is recommended to indicate the reason.

15. The term “significant trend” is an oxymoron. A better, though still questionable term would be “non-significant trend” or “trend towards significance”. Please be true to the data and use terms such as that, even if questionable, do not blatantly misrepresent the results.

16. The term “reward-related regions” might mean different things to different readers. Please be more specific as to which specific structures are encompassed by this term, providing a reference if needed.

17. This sentence is not clear but seems key: ‘Specifically, cardiac and gastric interoception may be encoded in the brain regions that subserve the functional roles of each interoception’. Please revise.

18. Likewise, please be clearer here: ‘To compare the signal strength in the insula subregions between cardiac and gastric interoception, we extracted the individual signal strength as beta weights for each condition. Then, we performed an analysis of variance on these values with the condition and anatomical location as the within-factors, independently for each hemisphere’. Please revise, and explain: what is the purpose of this analysis? And why it was performed in such a manner? One aspect of the article that needs to be improved is that the rationale for many of the steps is missing. For instance, the ‘why’ the paradigm and analyses included needs to be further expanded.

19. We recommend including a brief mention to constraints of generality proposed by Simons et al., 2017: https://journals.sagepub.com/doi/10.1177/1745691617708630. Also, do the authors think that awareness of interoceptive signals could drive active sensing, i.e., non-time locked processing of stimuli? see e.g., https://www.biorxiv.org/content/10.1101/2022.02.10.479959v1.abstract. This should be touched upon in the discussion.

20. How did the authors decide the segmentation of the insula? (rationale, references)

References

Khalsa, S. S., Adolphs, R., Cameron, O. G., Critchley, H. D., Davenport, P. W., Feinstein, J. S., ... & Zucker, N. (2018). Interoception and mental health: a roadmap. Biological psychiatry: cognitive neuroscience and neuroimaging, 3(6), 501-513.

Rebollo, I., & Tallon-Baudry, C. (2022). The sensory and motor components of the cortical hierarchy are coupled to the rhythm of the stomach during rest. Journal of Neuroscience, 42(11), 2205-2220.

## Author Response

Letter to the reviewers:

We are most thankful for the reviewer’s helpful and insightful comments. We have considered all the comments the reviewer provided, which greatly improved our original manuscript. Red-colored text in the manuscript indicates modifications. Our item-by-item responses to the reviewer’s comments are as follows.

1. The term interoception is used too loosely and is difficult to interpret. While the authors state in the introduction that they refer to ‘the awareness of signals that arise from within the body as interoception’, in the rest of the manuscript they use vague terms such as ‘cardiac and gastric interoception’, ‘different interoceptions’, etc. What type of interoception are the authors measuring with the task? How does the task relate to interoceptive attention, sensitivity, subjective awareness or signal intensity? Please refer to recent literature on cardiac interoception (e.g., Khalsa et al 2018) and, once the authors have settled on a specific interpretation of the term, they should use it consistently throughout the manuscript.

Response to Comment 1.

We have revised the whole manuscript to use the term ‘interoception’ consistently. Particularly, we first defined it as the phenomenal experience of one’s own bodily signals; moreover, we specified the definition of cardiac interoception and gastric interoception in the first paragraph because the present study focused mainly on the two. In addition, we introduced our experimental design-asking participants to direct their attention to bodily sensations-as interoceptive attention paradigm that has been used to investigate the neural activation of interoception. We believe that readers would easily understand what aspect of interoception we are studying and what task was chosen to investigate it. The revised sentences in the introduction are as follows:

(Page 2, Line 2)

Subjective experiences of one’s internal bodily state are referred to as interoception or interoceptive awareness (Brewer et al., 2021; Khalsa et al., 2018). Such senses indicate ongoing physiological changes, guiding agents in adaptive behavior (Paulus et al., 2019; Quigley et al., 2021). In this paper, we use the term interoception to describe the phenomenal experience of bodily states.

(Page2, Line 11)

We thus used functional magnetic resonance imaging (fMRI) to focus on the brain activation of cardiac interoception (i.e., awareness of one’s heartbeat sensation) and gastric interoception (awareness of the stomach sensation) that have been suggested to serve different functional roles.

(Page 3, Line 6)

Another way to map interoception is to use an interoceptive attention paradigm-asking participants to direct their attention toward sensations originating from within the body. Importantly, although exteroceptive information such as cutaneous sensations may contribute to interoception (Khalsa et al., 2009), interoceptive attention has been found to elicit a robust activation of visceral sensory areas, the insula (Haruki and Ogawa, 2021; Klabunde et al., 2019; Wiebking et al., 2014). Particularly, researchers have consistently associated the activation in the right anterior insula with interoceptive attention (Simmons et al., 2013; Zaki et al., 2012) and objective accuracy of cardiac interoception (Caseras et al., 2013; Critchley et al., 2004; Pollatos et al., 2007).

(Page 3, Line 18)

Previous studies using the interoceptive attention paradigm suffer from the limitation of having extensively used heartbeat attention (i.e., cardiac interoception) over other modalities of interoception. Even when the attentional focus on gastric interoception was deployed, direct comparison within the modality of interoception has not been discussed (DeVille et al., 2020; Kerr et al., 2016; Simmons et al., 2013). Therefore, it is still unclear whether neural encodings of the various interoception differ, limiting the theoretical advances in this field. To address this issue, we directly compared the neural activation for cardiac and gastric interoception using the interoceptive attention paradigm in a healthy population.

2. The authors asked participants to attend to sensations of their heart and stomach. While these sensations might originate within the body, the process of such information might reach participants’ sensorirum/awareness through exteroceptive modalities. For instance, heartbeats produce a pulsatile sensation that can be felt on the chest over the skin (a cutaneous stimulation). See for instance the studies reviewed here: https://www.sciencedirect.com/science/article/pii/S0010945222000685 (second paragraph in the introduction and those also reported to show small movements concomitant to heartbeats), see also: https://www.nature.com/articles/nn.2411. Hence, while changes inside the body are interoceptive in origin and principle, conscious awareness of these changes might entail exteroceptive processing. This idea should be more clearly stated in the manuscript.

Response to Comment 2.

We strongly agree that exteroceptive processing such as cutaneous sensations would affect interoceptive awareness or interoception. However, we would consider that the issue might be out of scope from the present study. The reason is that we focused on an awareness of one’s ongoing bodily states (what we called interoception), whether it arises from a particular viscera or from several sources including skins or respiratory tracts. Although we asked participants to focus on their internal sensations but not the skin, we could not completely rule out a possibility that participants felt the heartbeat sensation from their skins as the reviewer pointed. Yet, we consider that they were most likely aware of cardiac or gastric interoception during the task; we tried to measure the brain activation related to the awareness but not a pure visceroception. Anyway, we sincerely appreciate the reviewer’s comment that interoception could arise from not only the interoceptive signals but from cutaneous sensations; we thus revised the manuscript to reflect the reviewer’s suggestion. If we were misunderstanding the reviewer’s intention, we appreciate it if the reviewer specified the point of revision. Our revised sentence is as follows:

(Page 3, Line 6)

Another way to map interoception is to use an interoceptive attention paradigm-asking participants to direct their attention toward sensations originating from within the body. Importantly, although exteroceptive information such as cutaneous sensations may contribute to interoception (Khalsa et al., 2009), interoceptive attention has been found to elicit a robust activation of visceral sensory areas, the insula (Haruki and Ogawa, 2021; Klabunde et al., 2019; Wiebking et al., 2014).

(Page 19, Line 19)

On a related note, previous studies have suggested that external processes such as cutaneous sensations could affect interoception (particularly in the cardiac domain) (Khalsa et al., 2009). Although we asked participants to focus on their internal sensations, we could not completely rule out the possibility that participants felt the heartbeat sensation from their skins. However, in the first place, interoception may not arise solely from a single visceral sensation- as when heartbeat perception is felt as the contraction of blood vessels and beating of the heart, or hunger is mediated by both the physical contents of the stomach and a chemical signal. If so, researcher may benefit from isolating the neural substrates of cardiac and gastric interoception localized in the present study from a visceral topography to reveal the process of (visceral) multisensory integration.

3. Readers might appreciate seeing the difference in behavioral responses for cardiac, gastric and visual task in a figure. Are they statistically different? These results are not reported and are important to interpret brain data. Please provide descriptive and inferential statistics for behavioral data in full (see also point 8). Also, the cardiac signal is typically felt more strongly than gastric sensations during rest. Without an external manipulation of gastric and cardiac signals, the difference in result could reflect the difference in signal strength, attentional demand, fatigue and “on task” vs “off task”. Regarding the limitations of the task, they should be addressed more lengthly in the discussion. What are the task confounds, which other variables could be measuring?

Response to Comment 3.

As the reviewers suggested, we have provided the graph showing the distribution of raw behavioral data (Figure 3B) and the table showing detailed the detail of our statistical inference (Table 1) in addition to our original text-based reports. There was a statistically significant difference in subjective reports between heartbeat and stomach sensation. Thus, we performed a multiple regression analysis that excludes the effect of subjective reports on the brain activation, revealing that the brain activation of interoceptive attention was almost unaffected by individual differences in behavioral data (please see Figure 3C). Moreover, we have enriched discussion about the limitation of the present experiment. The revised sentences are as follows:

(Page 9, line 4)

Moreover, we performed a group-level analysis of covariance (ANCOVA) to exclude the effect of the subjective ratings of stimulus intensity on brain activation. This was because we considered that the differences in the subjective intensity may affect the brain activation pattern, independently of the object of attentional focus. Using the image of interoception contrasted to the visual control, we modeled individual subjective ratings for each condition, averaged for five runs, as covariates. Then, ANCOVA excluding the effects of subjective rating as nuisance was performed. By doing so, we assessed the brain activation of cardiac interoception contrasted to visual attention without the effect of individual differences in subjective heartbeat intensity and gastric interoception contrasted to visual without the subjective intensity of stomach sensation.

(Page 13, Line 4)

Furthermore, we confirmed that the brain activation elicited by cardiac and gastric interoception was almost unaffected by differences in subjective stimulus intensity by performing a multiple regression analysis excluding the effects of subjective ratings of stimulus intensity. The results of the multiple regression were fairly comparable to the brain activation obtained in cardiac and gastric interoception contrasted to visual control (Figure 3C). There was no brain activation covarying with the subjective intensity rating even at a more moderate threshold (cluster-level p < .05, uncorrected). All results are reported with a voxel-level threshold of p < .001 (uncorrected) with cluster size correction for p values < .05 (FWE).

(Page 19, Line 3)

Another possible limitation is that the difference in task difficulty in each condition could affect the results. Although we tried to make the difficulty comparable among each condition, there was a significant effect of the task condition on the subjective intensity of stimulus-subjective intensity of the visual stimulus was rated the highest while that of the stomach sensation the lowest of the three. We thus performed another analysis that excluded the effect of the subjective rating of stimulus intensity, indicating that the brain activation was almost unaffected by individual differences in the subjective rating. The results support our original ideas that the present brain activation in cardiac and gastric interoception was elicited by the object (the heartbeat and stomach sensation) of attentional focus not the difference in task difficulty. Recording a trial-by-trial fluctuation of the subjective intensity of stimulus would strengthen the present findings that the subjective differences in interoception are encoded differently in the brain.

4. There is no analysis comparing with the visual condition, why is this so? The visual condition is a valid control that does not seem to be used at all. At the very least, the authors should report results of the contrasts gastric > visual and cardiac > visual as well as their reverse for brain data and, as pointed out in the previous comment, report the full behavioral results. Furthermore, in MVPA analyses the authors could identify insula regions associated with cardiac and gastric interoception over and above the visual control condition.

Response to Comment 4.

In our original manuscript, we did not report results contrasting interoception to visual control because we focused on the difference in brain activation between cardiac and gastric interoception. We have provided the results of the visual contrasts as the reviewer suggested (Figure 3A, 3B), which greatly improved the manuscript by showing the activation similarity between cardiac and gastric interoception. We have also provided the results of direct comparison of the brain activation in interoception and exteroception as extended data (Figure 4-1). However, we could not perform MVPA that distinguished the brain activation pattern between interoception and exteroception because of the differences in the number of trials (30 trials for interoception and 15 trials for exteroception per participant) which would result in providing a biased classifier. The revised sentences in the main text are as follows:

(Page 8, Line 16)

We first evaluated the brain regions activated in each condition (heart attention, stomach attention, and visual attention) using a generalized linear model (GLM). Individual-level GLM included three regressors of interest for each condition as a separate box-car function that was convolved with the canonical hemodynamic response function. The rest period was used as a baseline. To reduce motion-related artifacts, six motion parameters were included as nuisance regressors. By combining the three conditions, we obtained six contrast images (heart attention compared to stomach attention, heart compared to visual, stomach compared to visual, and their opposite contrasts) for each participant. We then performed a group-level random effects analysis for these images using a one-sample t-test. Through these statistical inferences, we directly compared the brain activation for each condition across the whole brain.

(Page 11, Line 7)

Furthermore, we tested whether averaged activation for interoception and exteroception differed in the insula using a similar procedure in the case of cardiac and gastric interoception. We extracted mean signal changes for interoception, the averaged activation across heart and stomach attention, and exteroception (visual attention) in each ROI. Then, repeated-measures ANOVA with the condition (interoception and exteroception) and the anatomical location as within-factors was performed separately for each hemisphere.

(Page 12, Line 18)

Moreover, by comparing cardiac and gastric interoception to the visual control condition, we found that cardiac and gastric interoception activated similar brain regions, including the insula, frontal operculum, parietal operculum, middle cingulate, and supplementary motor area; the results were highly comparable to previous studies (Haruki and Ogawa, 2021; Simmons et al., 2013; Tan et al., 2018). A notable exception was that the hippocampus and medial visual areas were activated only in gastric interoception (Figure 3A). The visual control condition contrasted to cardiac and gastric interoception activated brain regions critical for visual attention, such as the middle frontal gyrus, superior parietal lobule, posterior thalamus (geniculate nucleus), and lateral visual association area (Dosenbach et al., 2007; Mayer et al., 2007), supporting the validity of our experimental design (Figure 3A).

(Page 14, Line 19)

Furthermore, we found that a differences in averaged activation between interoception and exteroception; the ANOVA on activations in the left insula revealed that significant effects of anatomical location (F5, 150 = 52.71, p < .001, ηp = .64) and interaction between condition and location (F5, 150 = 13.45, p < .001, ηp = .31) (Extended Data Figure 4-1). Post-hoc analysis showed that the left MSG and PSG were more activated by interoceptive attention (F1, 30 = 7.38, p = .011, ηp = 20; F1, 30 = 18.70, p < .001, ηp = .38, respectively). The ANOVA on the right insula activation revealed significant effects of anatomical location (F5, 150 = 41.02, p < .001, ηp = .58) and interaction between condition and location (F5, 150 = 12.23, p < .001, ηp = .28) as well. We found that exteroceptive attention more activated the right ASG than interoceptive attention by post-hoc analysis (F1, 30 = 10.28, p = .003, ηp = .26).

(in Figure caption)

As extended data, we performed a similar analysis focusing the subdivisions of the insula for averaged brain activation in interoception and exteroception (Extended Data Figure 4-1).

5. The MVPA analyses should be explained more clearly in both the results and methods sections, as at present it is unclear what the procedure and rationale of the analysis is. What conditions, exactly, were compared in each step, how, and why?

Response to Comment 5.

We have provided a detailed explanation about MVPA as the reviewer suggested. In particular, we have enriched the procedure of training and testing. We believe that the revision made the study much more convincing. The revised sentences are as follows:

(Page 9, Line 19)

We tested whether the subdivisions of the insula, the primary interoceptive cortex that receives the initial cortical input of visceral signals (Craig, 2002), had a different representation for cardiac and gastric interoception at the voxel level. In particular, we first performed MVPA that allows us to investigate more sophisticated neural representation compared with conventional analysis (Norman et al., 2006), suspecting that conventional analysis might fail to detect the activation difference in the insula.

(Page 10, Line 10)

MVPA for cardiac and gastric interoception was performed with a two-class classifier based on a linear support vector machine (SVM) implemented in LIBSVM (http://www.csie.ntu.edu.tw/∼cjlin/libsvm/). We first created another individual-level GLM apart from the whole-brain analyses that included 15 task trials as independent regressors per run with six motion parameters as nuisance regressors. By doing so, we obtained parameter estimates of all voxels in each ROI for a total of 75 trials per participant, labeling them as heart, stomach, or visual, depending on the condition of each trial. We then trained SVM to classify the identity of the brain activation pattern of cardiac and gastric interoception using these parameter estimates as inputs to the SVM. Individual-level classification accuracy was estimated with a five-fold “leave-one-out” cross-validation to avoid overfitting. This procedure uses inputs in four runs as training data and inputs in one remaining run as test data to be classified, which was repeated five times for all possible combinations. The averaged classification accuracy across five repetitions of the tests was computed for each ROI for each participant independently. We used a default hyperparameter (a fixed regularization parameter C = 1). Parameter estimates of the trial were not used as input to the classifier. One-sample t-tests were performed to test whether the activation patterns for cardiac and gastric interoception in each subregion were classifiable using SVM. That is, we tested whether the computed classification accuracy was higher than the chance level (50%) at the group level, separately for each ROI.

(Page 13, Line 14)

We performed MVPA using an SVM classifier that distinguished the activation patterns in the insula between cardiac and gastric interoception. Activation patterns in the anatomical subregions of the insula (Faillenot et al., 2017, Figure 4A) were used as inputs to the SVM. We found that classification accuracy in the left PSG, which corresponds to the dorsal middle insula, was significantly higher than the chance level (50%) (mean classification accuracy = 56.32, t30 = 3.39, corrected p-value for FDR = .024), suggesting that the region had distinct representation for cardiac and gastric interoception. The right ASG (mean = 53.48, t30 = 2.56, corrected p = .089), left ASG (mean = 54.26, t30 = 2.28, corrected p = .089), and left MSG (mean = 53.42, t30 = 2.34, corrected p = .089) showed a marginally significant classification accuracy above the chance level; all of them would correspond to the dorsal mid-anterior insula. The other subdivisions of the insula such as posterior or ventral subdivisions showed no significant classification accuracy above the chance level (corrected ps > .27) (Figure 4B).

6. Direct heart rate or gastric cycle measurements would have greatly strengthened the study, as it would have allowed to measure not only where subjects’ attention is directed, but also how this relates to objective changes in cardiac or gastric rhythms (e.g., whether higher intensity ratings correspond with accelerations in heart rate). Are these measurements available? If not, this should be discussed as a major limitation, and studies using objective measurements should be clearly distinguished from the present one in the introduction (see also point 1).

Response to Comment 6.

Unfortunately, we were unable to record the physiological variables due to a technical issue, limiting the present results as the reviewer pointed. We thus have revised the manuscript to show that the present study used an interoceptive attention paradigm, apart from the objective measurements of physiological recording. Moreover, we have added the discussion about the lack of physiological recordings. The revised sentences are as follows:

(Page 3, Line 6)

Another way to map interoception is to use an interoceptive attention paradigm-asking participants to direct their attention toward sensations originating from within the body. Importantly, although exteroceptive information such as cutaneous sensations may contribute to interoception (Khalsa et al., 2009), interoceptive attention has been found to elicit a robust activation of visceral sensory areas, the insula (Haruki and Ogawa, 2021; Klabunde et al., 2019; Wiebking et al., 2014). Particularly, researchers have consistently associated the activation in the right anterior insula with interoceptive attention (Simmons et al., 2013; Zaki et al., 2012) and objective accuracy of cardiac interoception (Caseras et al., 2013; Critchley et al., 2004; Pollatos et al., 2007).

(Page 3, Line 18)

Previous studies using the interoceptive attention paradigm suffer from the limitation of having extensively used heartbeat attention (i.e., cardiac interoception) over other modalities of interoception. Even when the attentional focus on gastric interoception was deployed, direct comparison within the modality of interoception has not been discussed (DeVille et al., 2020; Kerr et al., 2016; Simmons et al., 2013). Therefore, it is still unclear whether neural encodings of the various interoception differ, limiting the theoretical advances in this field. To address this issue, we directly compared the neural activation for cardiac and gastric interoception using the interoceptive attention paradigm in a healthy population.

(Page 19; Line 13)

Unfortunately, we did not record any physiological data during the fMRI scanning, potentially limiting the current study. Simultaneous recording of electrocardiogram (ECG) and electrogastrogram would address the interaction between brain activation and physiological change evoked by interoceptive attention. But, we consider that the lack of physiological recording does not challenge the validity of the present study because past research found that cardiac parameters (heart rate and ECG amplitude) did not differ by directing attention between interoception and exteroception (Petzschner et al., 2019).

7. The authors did not analyze how changes in bold signal vary as a function the subjects’ reports of sensations intensity. We suggest the authors to run both within-subject (parametric modulation with trial-wise intensity ratings) and between-subject (regression with mean intensity reports) analyses, reporting the results separately for each condition, and comparing the conditions between them. These analyses will strongly improve the paper.

Response to Comment 7.

We performed a regression analysis that had the mean intensity report as covariate of interest as the reviewer suggested, but we found no significant activation across the brain. Conversely, we confirmed that the brain activation was not almost affected by the subjective intensity of each stimulus by performing a group-level analysis of covariance (Figure 3C), improving our manuscript a lot. Unfortunately, we cannot run a parametric modulation because we did not require a trial-by-trial intensity report. This was because we were afraid that trial-by-trial reports could contaminate the brain activation during the attentional phase (e.g., preparation for rating score or motor response). We have revised the manuscript to describe the procedure of multiple regression analysis and enriched the limitation section as follows:

(Page 9, Line 4)

Moreover, we performed a group-level analysis of covariance (ANCOVA) to exclude the effect of the subjective ratings of stimulus intensity on brain activation. This was because we considered that the differences in the subjective intensity may affect the brain activation pattern, independently of the object of attentional focus. Using the image of interoception contrasted to the visual control, we modeled individual subjective ratings for each condition, averaged for five runs, as covariates. Then, ANCOVA excluding the effects of subjective rating as nuisance was performed. By doing so, we assessed the brain activation of cardiac interoception contrasted to visual attention without the effect of individual differences in subjective heartbeat intensity and gastric interoception contrasted to visual without the subjective intensity of stomach sensation. Furthermore, we explored brain activations varying as a function of the subjective intensity; a regression analysis with the subjective intensity as a covariate of interest was performed using the same model used in ANCOVA. The voxel-level threshold was set to p < .001 (uncorrected), and the cluster-level threshold was set to p < .05, corrected for family-wise error (FWE).

(Page 13, Line 4)

Furthermore, we confirmed that the brain activation elicited by cardiac and gastric interoception was almost unaffected by differences in subjective stimulus intensity by performing a multiple regression analysis excluding the effects of subjective ratings of stimulus intensity. The results of the multiple regression were fairly comparable to the brain activation obtained in cardiac and gastric interoception contrasted to visual control (Figure 3C). There was no brain activation covarying with the subjective intensity rating even at a more moderate threshold (cluster-level p < .05, uncorrected).

(Page 19, Line 3)

Another possible limitation is that the difference in task difficulty in each condition could affect the results. Although we tried to make the difficulty comparable among each condition, there was a significant effect of the task condition on the subjective intensity of stimulus-subjective intensity of the visual stimulus was rated the highest while that of the stomach sensation the lowest of the three. We thus performed another analysis that excluded the effect of the subjective rating of stimulus intensity, indicating that the brain activation was almost unaffected by individual differences in the subjective rating. The results support our original ideas that the present brain activation in cardiac and gastric interoception was elicited by the object (the heartbeat and stomach sensation) of attentional focus not the difference in task difficulty. Recording a trial-by-trial fluctuation of the subjective intensity of stimulus would strengthen the present findings that the subjective differences in interoception are encoded differently in the brain.

8. On a related note, the results of the linear mixed effects models should be reported in full, including degrees of freedom, regression coefficients, and p-values. Also, please ensure that the random effects structure is maximally specified: https://www.ncbi.nlm.nih.gov/pmc/articles/PMC3881361/ Furthermore, raincloud plots should be used to display intensity ratings per condition (as suggested in point 3).

Response to Comment 8.

We have we reanalyzed our linear mixed effects model to assure our models had the maximal random structure and provided a new table showing the full results of our linear mixed effects model analyses (Table 1). The distribution plot has been provided as a point plot with half violin plot (Figure 3B) as the present measure of seven-point Likert scale is not suitable for a stripplot or swarmplot. Although the results of reanalysis remained statistically significant and did not affect our discussion, the clarification of procedure improved the manuscript to be more convincing. The revised sentences describing the structure and advantage of our models are as follows:

(Page 8, Line 4)

We used R (version 4.0.3) for all our statistical inference except functional imaging data. First, we tested whether the subjective intensity of sensations differed between modalities (cardiac, gastric, and visual). The linear mixed-effects (LME) model analysis implemented in the lme4 package (Bates et al., 2015) was performed. With all the data obtained (N = 465, 3 trial types for 5 runs for 31 participants) as a dependent variable, we modeled the type of sensation as the fixed effect. Random intercept and slope for the effects of participants and random intercept for the runs were modeled as random effects, ensuring the maximal random structure for our models (Barr et al., 2013). The LME enabled us to avoid averaging the values across five runs compared to the traditional analysis of variance (ANOVA) (Baayen et al., 2008). The parameter was estimated using the restricted maximum likelihood method, with the degrees of freedom estimated using the Satterthwaite method.

9. The overlap with the gastric network is not actually quantified, please provide a standard metric in this regard. Also, the authors should considering using the overlays from the most recent version of the gastric network from Rebollo & Tallon-Baudry (2022) https://identifiers.org/neurovault.collection:9985

Response to Comment 9.

As the reviewer suggested, we have recreated the figure showing the overlap between the latest version of gastric network (Rebollo & Tallon-Baudry, 2022) and the brain regions activated in gastric attention (Figure 2B).

10. Cardiac interoception. The article could benefit from some quantitative comparison with other studies of cardiac interoception. For instance, how the heart cluster relates to neurosynth studies of the term interoceptive (https://neurosynth.org/analyses/terms/interoceptive/) or heart (https://neurosynth.org/analyses/terms/heart/). Moreover, the scope of the discussion on cardiac interception should be limited. This is mainly because the limitations of the task in measuring more specific components of interoception. In particular, the fifth paragraph of the discussion should be restructured. The messages are difficult to extract and is generally difficult to read.

Response to Comment 10.

We have revised the paragraph discussing cardiac interoception as the reviewers suggested. We did not use the results of neurosynth meta-analysis because it appears to include many studies unrelated to the present study-as the word “heart” includes studies treating heartrate and “interoceptive” encompasses studies using an affective picture to present visually. Instead, we have added citations of studies using cardiac interoception and a systematic review focusing on the brain activation during cardiac interoception (Schulz, 2016). More important, we have cited studies that investigated the effects of right insula resection on cardiac interoception (Holtmann et al., 2022; Terasawa et al., 2021) and the effects of false cardiac feedback on insula activation (Gray et al., 2007; Kleint et al., 2015) to strengthen our discussion about the right insula dominance in cardiac interoception.

Furthermore, we have thoughtfully revised the fifth paragraph, following the reviewer’s concern. In the revised paragraph, we summarized our ROI analyses, suggesting a distinct brain activation pattern in the left middle insula. We then discussed the heterogenous role of the subdivisions of the insula based on the present results and previous findings.

The revised paragraphs are as follows:

(Page 16, Line 12)

Previously, heartbeat perception has been studied as a representative of interoception in general, suggesting the right anterior insula as the most relevant region for generating subjective experience of internal bodily states (Craig, 2009; Critchley and Garfinkel, 2017). We, however, found that cardiac interoception activated the right anterior insula more than gastric interoception, suggesting that the right anterior insula may dominantly code cardiac interoception, rather than the interoception arising from other sources (e.g., gastric). In line with this idea, the most consistent activation in the right insula has been found by a meta-analysis focusing on cardiac interoception (Schulz, 2016). Furthermore, previous studies have found a robust correlation between individual accuracy of cardiac interoception and heightened activation of the right anterior insula (Caseras et al., 2013; Critchley et al., 2004; Haruki and Ogawa, 2021; Pollatos et al., 2007), but this was not the case for awareness of breathing (Wang et al., 2019) or skin conductance (Baltazar et al., 2021). The activation dominance in the right anterior insula may be explained by its functional roles: people can notice increased arousal with cardiac interoception (Paulus et al., 2019). The right insula appears essential to perceive arousal as the resection of the region causally diminishes physiological and emotional arousal (Holtmann et al., 2022; Terasawa et al., 2021). Moreover, presenting accelerated cardiac feedback increases perceived physiological arousal (Story and Craske, 2008) and, more importantly, activates the right anterior insula (Gray et al., 2007; Kleint et al., 2015). These findings, in conjunction with the present results, imply that cardiac interoception closely involves physiological arousal and thus activates the right anterior insula by merely focusing on cardiac sensation. To sum up, the relationship between the right anterior insula and interoception appears stronger in cardiac interoception than in other modalities; such dominance of cardiac interoception may reflect the functional role of cardiac interoception that informs physiological arousal.

(Page 18, Line 20)

The present ROI analyses revealed that the insula had distinct representations for cardiac and gastric interoception. Only the right ASG and MSG, corresponding to the dorsal anterior insula, showed higher activation in cardiac than gastric interoception; in the right mid-posterior and left insula, there was no activation difference. Nevertheless, we identified the left PSG, corresponding to the dorsal middle insula, in different activation patterns for cardiac and gastric interoception. These results elaborate on how the human insula represents interoception; in particular, we suggest the left middle insula as coding the viscera-specific interoception for the first time. Interestingly, the posterior insula did not show separable activation for cardiac and gastric interoception nor higher activation than baseline. Researchers have considered the posterior insula as “primary” interoceptive cortex as it receives an initial cortical input of visceral signals (Craig, 2002; Evrard, 2019). The current inactivity of the posterior insula may support its role that encodes an ongoing change of bodily signals (Craig et al., 2000; Meier et al., 2018), rather than a subjective awareness of bodily states. This is because, in the interoceptive attention task, participants must be aware of interoception without homeostatic perturbation. Together, our ROI analyses would support the gradual process of bodily signals in the insula along the posterior-anterior axis (Craig, 2009): the posterior codes physical changes of the signal while the subjective differences in interoception would be represented in the middle-anterior insula.

11. From a subject perspective, the authors should state that when attending to either stimuli (cardiac, gastric, visual), it is not possible to fully ignore sensations of the body. For instance, I could be attending to my stomach but noticing my heart. Please comment on this. Did the authors correct/dissociate for such an issue?

Response to Comment 11.

We agree with the reviewer’s comment that one may not successfully ignore sensations arising from other sources. We consider that people would get aware of interoception in the same manner in daily life; for example, hungry people might feel a slight thirst at the same time but feel stronger sensation of hunger. Therefore, we consider it might make little sense to discuss the “pure” cardiac interoception or gastric interoception in this sense. On related note, we asked participants to perform a practice trial before entering to MRI scanner to check whether participants successfully directed their attention to either heartbeat or stomach sensation depending on the instruction. We have added the sentence explaining the procedure, as follows:

(Page 6, Line 21)

Participants experienced a practice trial and its intensity report (for each condition) to learn how to perform the task and report the subjective intensity before entering in the MRI scanner. Through the practice trials, we verbally confirmed that participants successfully directed their attention to each sensation depending on the condition.

12. The authors did not report whether the number of trials and participants was appropriate and based upon prior evidence. The use of power calculation and/or citing papers with similar paradigms and analyses (as examples/guide) can aid to better buttress the design.

Response to Comment 12.

We have added explanations of our study design, particularly in relation to previous studies that used the interoceptive attention paradigm as well. The revised sentences are as follows:

(Page 5, Line 9)

The sample size deemed sufficient for determining reliable brain activation was calculated based on previous studies that have used a similar task for interoception (Haruki and Ogawa, 2021; Wiebking and Northoff, 2015).

(Page 6, Line 14)

An approximately 5.5 min run was repeated five times; thus, we obtained 25 data points (125 volumes) for each condition per participant. This procedure was designed to include larger number of trials compared with previous studies (DeVille et al., 2020; Kerr et al., 2016) because we needed sufficient number of task trials to perform MVPA.

13. The last line of the first paragraph of the discussion is unclear, please reformulate

Response to Comment 13.

We have reformulated the first paragraph of the discussion as the reviewers suggested. The revised paragraph is as follows:

(Page 16, line 2)

Our results empirically demonstrate that cardiac and gastric interoception have similar but distinct neural substrates. Direct comparison of brain activation revealed that cardiac and gastric interoception activated similar brain regions including the insula, frontal operculum, parietal operculum, middle cingulate, and supplementary motor area, when contrasted to the control visual condition. However, compared to gastric interoception, cardiac interoception activated the right anterior insula extending to the frontal operculum more; gastric interoception enhanced activations in the occipitotemporal visual cortices, bilateral primary motor, primary somatosensory, left orbitofrontal, and bilateral posterior hippocampus. Moreover, our MVPA-based ROI analyses revealed that the left middle-anterior insula had a distinct neural representation for cardiac and gastric interoception.

14. If possible the authors should share the data and code in an open repository. Please add the statement in the article about data availability, and if it is not possible, it is recommended to indicate the reason.

Response to Comment 14.

We have added the Data availability statement, providing all data used in our Figures and Tables available. The sentences are as follows:

(on title page)

Data availability statement

The .mat files containing the group-level brain activation, the results of our ROI analyses, and the behavioral data are available at https://osf.io/xxxxx. The computer codes are available from the corresponding author upon reasonable request.

15. The term “significant trend” is an oxymoron. A better, though still questionable term would be “non-significant trend” or “trend towards significance”. Please be true to the data and use terms such as that, even if questionable, do not blatantly misrepresent the results.

Response to Comment 15.

We appreciate that the reviewers pointed out our problematic word usage. We have replaced the word with “marginally significant”, which is most commonly used (Otte et al., 2022). The revised sentences are as follows:

(Page 13, Line 19)

The right ASG (mean = 53.48, t30 = 2.56, corrected p = .089), left ASG (mean = 54.26, t30 = 2.28, corrected p = .089), and left MSG (mean = 53.42, t30 = 2.34, corrected p = .089) showed a marginally significant classification accuracy above the chance level; all of them would correspond to the dorsal mid-anterior insula. The other subdivisions of the insula such as posterior or ventral subdivisions showed no significant classification accuracy above the chance level (corrected ps > .27) (Figure 4B).

(Page 14, Line 15)

Post-hoc analysis of the left ROIs revealed that the ALG (corresponding to the dorsal posterior insula) showed a marginally significant activation higher in gastric than cardiac interoception (F1, 30 = 3.83, corrected p = .059). Post-hoc analysis of the right ROIs also revealed that the right ASG and MSG exhibited higher activation in cardiac than gastric interoception (F1, 30 = 5.93, corrected p = .021; F1, 30 = 6.57, corrected p = .016, respectively) while marginally significant activation higher in gastric interoception was found in the ALG (F1, 30 = 4.13, corrected p = .051).

16. The term “reward-related regions” might mean different things to different readers. Please be more specific as to which specific structures are encompassed by this term, providing a reference if needed.

Response to Comment 16.

We have removed the phrase from the manuscript as the reviewer suggested. Instead, we have specified the roles of activated brain regions. The revised sentences are as follows:

(Page 18, Line 2)

The reason for the involvement of the visual area may be that gastric interoception is closely related to foraging and feeding behavior that requires the integration of visuospatial information with energy status and motor coordination (Kanoski and Grill, 2017; Rebollo et al., 2018). Presumably, hungry people would change their sensory sampling and behavior in the external world to maximize the chance of food consumption.

(Page 18, Line 12)

Furthermore, the role of the hippocampus in controlling food intake and regulating energy status has received considerable attention in the past few years (Quigley et al., 2021; Suarez et al., 2019); recent evidence indicates that the human hippocampus encodes ongoing nutritional states in response to food cues (Jones et al., 2021).

17. This sentence is not clear but seems key: ‘Specifically, cardiac and gastric interoception may be encoded in the brain regions that subserve the functional roles of each interoception’. Please revise.

Response to Comment 17.

As the reviewer suggested, we have revised the sentence to clearly summarize our discussion. In addition, we have revised the whole abstract to make it clearer and more convincing as follows:

(Page 1, Line 2)

Interoception, or an awareness of the internal body state, guides agents in adaptive behavior by informing them of ongoing bodily signals, such as heart rate or energy status. However, it is still unclear whether the human brain represents the differences in the subjective experience of interoception differently. Hence, we directly compared the neural activation for cardiac (awareness related to heartbeats) and gastric (awareness related to the stomach) interoception in the same population (N = 31). Participants were asked to focus on their heart and stomach sensations to be aware of interoception in a magnetic resonance imaging scanner. The results indicated that neural activation underlying gastric interoception encompassed larger brain regions, including the occipitotemporal visual cortices, bilateral primary motor cortex, primary somatosensory cortex, left orbitofrontal cortex, and bilateral hippocampal regions. Cardiac interoception, however, selectively activated the right anterior insula extending to the frontal operculum more compared to gastric interoception. Moreover, our detailed analyses focusing on the insula, the most relevant region for interoception, revealed that the left dorsal middle insula encoded cardiac and gastric interoception in different activation patterns but not the posterior insula. Our results demonstrate that cardiac and gastric interoception have distinct neural substrates; in particular, the selective brain activation may reflect differences in the functional roles of cardiac and gastric interoception.

18. Likewise, please be clearer here: ‘To compare the signal strength in the insula subregions between cardiac and gastric interoception, we extracted the individual signal strength as beta weights for each condition. Then, we performed an analysis of variance on these values with the condition and anatomical location as the within-factors, independently for each hemisphere’. Please revise, and explain: what is the purpose of this analysis? And why it was performed in such a manner? One aspect of the article that needs to be improved is that the rationale for many of the steps is missing. For instance, the ‘why’ the paradigm and analyses included needs to be further expanded.

Response to Comment 18.

We have added reasons and rationales of the present experimental and analytical protocol. They have been noted in the introduction, methods, and results. As the reviewers suggested, we believe that the manuscript became much more convincing. The revised sentences are as follows:

(Page 3., Line 18)

Previous studies using the interoceptive attention paradigm suffer from the limitation of having extensively used heartbeat attention (i.e., cardiac interoception) over other modalities of interoception. Even when the attentional focus on gastric interoception was deployed, direct comparison within the modality of interoception has not been discussed (DeVille et al., 2020; Kerr et al., 2016; Simmons et al., 2013). Therefore, it is still unclear whether neural encodings of the various interoception differ, limiting the theoretical advances in this field. To address this issue, we directly compared the neural activation for cardiac and gastric interoception using the interoceptive attention paradigm in a healthy population. We hypothesized that cardiac and gastric interoception would elicit distinct neural activations, which would be modulated depending on their functional role. That is, cardiac interoception might activate the regions underlying physiological arousal; brain areas that modulate feeding and foraging behavior might show enhanced activation in gastric interoception. Moreover, we considered that the insula, the most relevant region for interoception (Craig, 2009; Critchley and Harrison, 2013), would show a subregion-specific representation of cardiac and gastric interoception. To test this idea, we performed a region of interest (ROI) analysis focusing on the insula by combining multivoxel pattern analysis (MVPA) with a basic comparison of neural activation.

(Page 8, Line 16)

We first evaluated the brain regions activated in each condition (heart attention, stomach attention, and visual attention) using a generalized linear model (GLM). Individual-level GLM included three regressors of interest for each condition as a separate box-car function that was convolved with the canonical hemodynamic response function. The rest period was used as a baseline. To reduce motion-related artifacts, six motion parameters were included as nuisance regressors. By combining the three conditions, we obtained six contrast images (heart attention compared to stomach attention, heart compared to visual, stomach compared to visual, and their opposite contrasts) for each participant. We then performed a group-level random effects analysis for these images using a one-sample t-test. Through these statistical inferences, we directly compared the brain activation for each condition across the whole brain.

(Page 9, Line 19)

We tested whether the subdivisions of the insula, the primary interoceptive cortex that receives the initial cortical input of visceral signals (Craig, 2002), had a different representation for cardiac and gastric interoception at the voxel level. In particular, we first performed MVPA that allows us to investigate more sophisticated neural representation compared with conventional analysis (Norman et al., 2006), suspecting that conventional analysis might fail to detect the activation difference in the insula.

(Page 11, Line 6)

We also compared the difference in the averaged activation in the ROIs among each condition because we suspected that the classification accuracy calculated by MVPA could merely reflect the mean signal change in the ROIs. First, the mean signal changes in each ROI were extracted for each participant, separately for the cardiac and gastric attention condition. Then, we performed a repeated-measures ANOVA on these values with the condition (cardiac and gastric) and anatomical location (the ASG, MSG, PSG, AIC, ALG, and PLG) as within-factors, independently for each hemisphere. Multiple comparison correction for post-hoc analyses was performed using Shaffer’s modified sequentially rejective Bonferroni procedure implemented in R. The combined use of MVPA and direct comparisons of the averaged signal change allowed detailed investigation of how the human insula represents interoception specific to the cardiac and gastric domain.

19. We recommend including a brief mention to constraints of generality proposed by Simons et al., 2017: https://journals.sagepub.com/doi/10.1177/1745691617708630. Also, do the authors think that awareness of interoceptive signals could drive active sensing, i.e., non-time locked processing of stimuli? see e.g., https://www.biorxiv.org/content/10.1101/2022.02.10.479959v1.abstract. This should be touched upon in the discussion.

Response to Comment 19.

We have provided the Constraints of Generality section to show the characteristic of the participants as follows:

(on title page)

Constraints on Generality

All participants were healthy students at xxxxx University, native speakers of xxxxx.

Our answer to the question is yes. We believe that awareness of interoceptive signals, referred to as interoception in the manuscript, can drive the active sensing or sampling of sensory (exteroceptive) signals. When people are aware of interoception (e.g., thirst, hunger, palpitation, fever, and so on) in daily life, they probably have to change sensory sampling and behavior to address the physiological needs. This is we think the functional roles of interoception presented in the present manuscript. We have revised the introduction and discussion to reflect this idea as follows:

(Page 2, Line 5)

Examples of interoception changing the way agents sample sensory information and act in the external world are as follows: thirst compels agents to prioritize finding and drinking water, heartbeat perception signals physiological arousal and increases vigilance, and an empty stomach informs when and where to eat.

(Page 18, Line 2)

The reason for the involvement of the visual area may be that gastric interoception is closely related to foraging and feeding behavior that requires the integration of visuospatial information with energy status and motor coordination (Kanoski and Grill, 2017; Rebollo et al., 2018). Presumably, hungry people would change their sensory sampling and behavior in the external world to maximize the chance of food consumption.

20. How did the authors decide the segmentation of the insula? (rationale, references)

Response to Comment 20.

We used the anatomical subdivisions of the insula (Faillenot et al., 2017) that were found to show a subregion-specific activation pattern for cardiac interoception (Haruki and Ogawa, 2021). We have clarified the ROI selection in the method section. The revised sentences are as follows:

(Page 9, Line 24)

The ROIs were defined as the anatomical subdivisions of the insula in Hammersmith brain atlases (Brain Development, www.brain-development.org). These images were constructed as a 3D probabilistic atlas using in vivo T1 MR images, including anatomical structures commonly seen in the human insula: the anterior short gyrus (ASG; the most dorsal anterior portion of the insula), middle short gyrus (MSG; the dorsal mid-anterior), posterior short gyrus (PSG; the dorsal mid-posterior), anterior inferior cortex (AIC; the ventral anterior), anterior long gyrus (ALG; the dorsal posterior), and posterior long gyrus (PLG; the ventral posterior) (Faillenot et al., 2017). Importantly, these ROIs have been confirmed to show a subregion-specific activation pattern for cardiac interoception (Haruki and Ogawa, 2021).

References

Brewer R, Murphy J, Bird G (2021) Atypical interoception as a common risk factor for psychopathology: A review. Neurosci Biobehav Rev 130:470-508.

Craig AD (2009) How do you feel - now? The anterior insula and human awareness. Nat Rev Neurosci 10:59-70.

Craig AD (2002) How do you feel? Interoception: the sense of the physiological condition of the body. Nat Rev Neurosci 6:655-666.

Critchley HD, Harrison NA (2013) Visceral Influences on Brain and Behavior. Neuron 77:624-638.

DeVille DC, Kuplicki R, Stewart JL, Paulus MP, Khalsa SS, Aupperle RL, Bodurka J, Cha YH, Feinstein J, Savitz JB, Victor TA (2020) Diminished responses to bodily threat and blunted interoception in suicide attempters. Elife 9:e51593.

Faillenot I, Heckemann RA, Frot M, Hammers A (2017) Macroanatomy and 3D probabilistic atlas of the human insula. Neuroimage 150:88-98.

Gray MA, Harrison NA, Wiens S, Critchley HD (2007) Modulation of emotional appraisal by false physiological feedback during fMRI. PLoS One 2.

Haruki Y, Ogawa K (2021) Role of anatomical insular subdivisions in interoception: Interoceptive attention and accuracy have dissociable substrates. Eur J Neurosci 53:2669-2680.

Holtmann O, Franz M, Mönig C, Tenberge JG, Schloßmacher I, Ivanova I, Preul C, Schwindt W, Melzer N, Miltner WHR, Straube T (2022) Lateralized deficits in arousal processing after insula lesions: Behavioral and autonomic evidence. Cortex 148:168-179.

Kanoski SE, Grill HJ (2017) Hippocampus Contributions to Food Intake Control: Mnemonic, Neuroanatomical, and Endocrine Mechanisms. Biol Psychiatry 81:748-756.

Kerr KL, Moseman SE, Avery JA, Bodurka J, Zucker NL, Simmons WK (2016) Altered insula activity during visceral interoception in weight-restored patients with anorexia nervosa. Neuropsychopharmacology 41:521-528.

Khalsa SS et al. (2018) Interoception and Mental Health: A Roadmap. Biol Psychiatry Cogn Neurosci Neuroimaging 3:501-513.

Khalsa SS, Rudrauf D, Feinstein JS, Tranel D (2009) The pathways of interoceptive awareness. Nat Neurosci 12:1494-1496.

Klabunde M, Juszczak H, Jordan T, Baker JM, Bruno J, Carrion V, Reiss AL (2019) Functional neuroanatomy of interoceptive processing in children and adolescents: a pilot study. Sci Rep 9.

Kleint NI, Wittchen HU, Lueken U (2015) Probing the interoceptive network by listening to heartbeats: An fMRI study. PLoS One 10:e0133164.

Norman KA, Polyn SM, Detre GJ, Haxby J V (2006) Beyond mind-reading : multi-voxel pattern analysis of fMRI data. Trends Cogn Sci 10:424-430.

Otte WM, Vinkers CH, Habets PC, van IJzendoorn DGP, Tijdink JK (2022) Analysis of 567,758 randomized controlled trials published over 30 years reveals trends in phrases used to discuss results that do not reach statistical significance. PLoS Biol 20:1-15.

Paulus MP, Feinstein JS, Khalsa SS (2019) An Active Inference Approach to Interoceptive Psychopathology. Annu Rev Clin Psychol 15:97-122.

Quigley KS, Kanoski S, Grill WM, Barrett LF, Tsakiris M (2021) Functions of Interoception: From Energy Regulation to Experience of the Self. Trends Neurosci 44:29-38.

Rebollo I, Devauchelle AD, Béranger B, Tallon-Baudry C (2018) Stomach-brain synchrony reveals a novel, delayed-connectivity resting-state network in humans. Elife 7:e33321.

Schulz SM (2016) Neural correlates of heart-focused interoception : a functional magnetic resonance imaging meta-analysis. Philos Trans R Soc B Biol Sci 371.

Simmons WK, Avery JA, Barcalow JC, Bodurka J, Drevets WC, Bellgowan P (2013) Keeping the body in mind: Insula functional organization and functional connectivity integrate interoceptive, exteroceptive, and emotional awareness. Hum Brain Mapp 34:2944-2958.

Terasawa Y, Motomura K, Natsume A, Iijima K, Chalise L, Sugiura J, Yamamoto H, Koyama K, Wakabayashi T, Umeda S (2021) Effects of insular resection on interactions between cardiac interoception and emotion recognition. Cortex 137:271-281.

Wiebking C, Duncan NW, Tiret B, Hayes DJ, Marjaǹska M, Doyon J, Bajbouj M, Northoff G (2014) GABA in the insula - a predictor of the neural response to interoceptive awareness. Neuroimage 86:10-18.

Wiebking C, Northoff G (2015) Neural activity during interoceptive awareness and its associations with alexithymia-An fMRI study in major depressive disorder and non-psychiatric controls. Front Psychol 6:1-16.
