# Supplementary figures and images for: Cardiac and Gastric Interoceptive Awareness Have Distinct Neural Substrates
Source: eNeuro. 2023 Jan 27;10(1):ENEURO.0157-22.2023. doi: 10.1523/ENEURO.0157-22.2023 (PMC9887674; doi:10.1523/ENEURO.0157-22.2023)

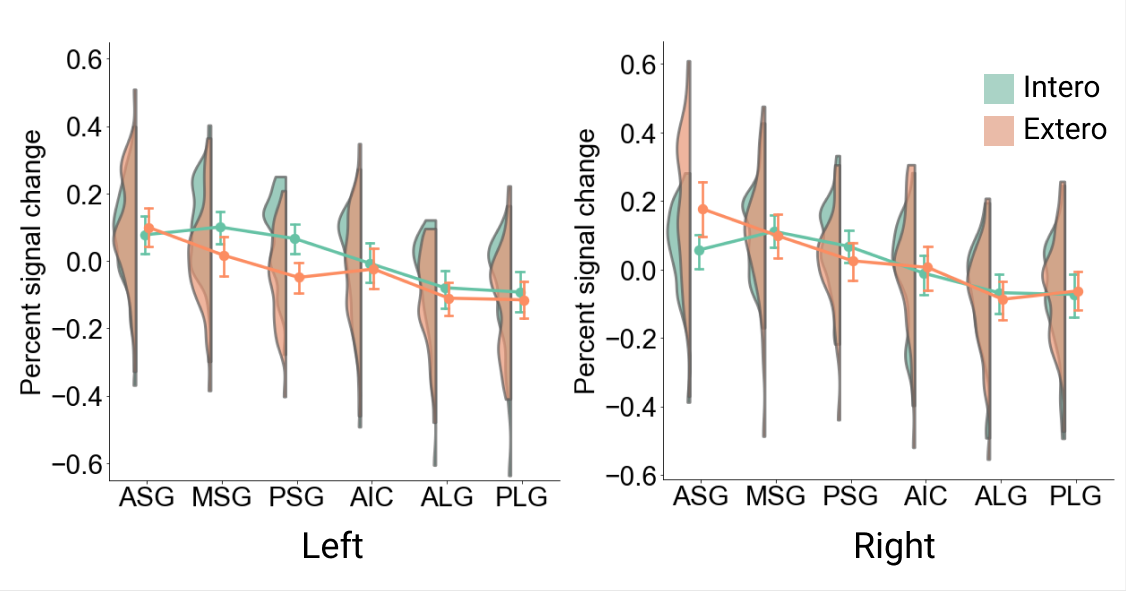

Supplement: Extended Data Figure 4-1 — Comparison of activations for interoceptive and exteroceptive attention in the subdivisions of the insula. The results of direct comparison of the signal strength between interoceptive (averaged for heartbeat attention and stomach) and exteroceptive (visual) attention are plotted. Post hoc analysis of repeated-measures AMOVA revealed that the right ASG showed significantly higher activation in exteroceptive than interoceptive attention, while the left MSG and PSG were activated more in interoceptive attention. The point plots represent the mean signal change, with 95% confidence intervals, while the half-violin plots represent the kernel density estimation (green for interoceptive attention, orange for exteroceptive attention). Download Figure 4-1, TIF file. [file enu-eN-NWR-0157-22-s01.tif]
